# Metabolomics profiling of pre-and post-anesthesia plasma samples of colorectal patients obtained via Ficoll separation

**DOI:** 10.1007/s11306-015-0832-5

**Published:** 2015-07-25

**Authors:** Veronica Ghini, Florian T. Unger, Leonardo Tenori, Paola Turano, Hartmut Juhl, Kerstin A. David

**Affiliations:** 1 CERM and Department of Chemistry, University of Florence, Florence, Italy; 2 Indivumed GmbH, Falkenried 88, 20251 Hamburg, Germany; 3 FiorGen Foundation, Via L. Sacconi 6, 50019 Sesto Fiorentino, Italy

**Keywords:** Metabolomics, Colorectal cancer, Metastatic colorectal cancer, Anesthesia, NMR

## Abstract

^1^H NMR spectroscopy was used to investigate the metabolic consequences of general anesthesia in the plasma of two groups of patients with diagnosis for non-metastatic colorectal cancer and metastatic colorectal cancer with liver-metastasis, respectively. Patients were treated with etomidate or propofol, two frequently used sedation agents. Plasma samples were obtained via Ficoll separation. Here, we demonstrated that this procedure introduces a number of limitations for NMR-based metabolomics studies, due to the appearance of spurious signals. Nevertheless, the comparison of the ^1^H NMR metabolomic profiles of patients treated with etomidate or propofol at equipotent dose ranges was still feasible and proved that both agents significantly decrease the plasma levels of several NMR-detectable metabolites. Consequently, samples collected during anesthesia are not suitable for metabolic profiling studies aimed at patient stratification, because interpersonal variability are reduced by the overall depression of metabolites levels. On the other hand, this study showed that plasma metabolomics could represent a valuable tool to monitor the effect of different sedation agents and/or the individual metabolic response to anesthesia, providing hints for an appropriate tuning of personalized sedation procedures. In our reference groups, the metabolomic signatures were slightly different in patients anesthetized with etomidate versus propofol. The importance of standardized collection procedures and availability of exhaustive metadata of the experimental design for the accurate evaluation of the significance of the observed changes in metabolites levels are critically discussed.

**Electronic supplementary material:**

The online version of this article (doi:10.1007/s11306-015-0832-5) contains supplementary material, which is available to authorized users.

## Introduction

The development of personalized medicine (determining an individual’s disease risk, prognosis, and therapeutic options) is fostered by high-throughput analysis of molecular biomarkers (DNA, RNA, protein or metabolites) in human biospecimens (Zatloukal and Hainaut 2010; Eckhart et al. 2012). Insufficient quality of such specimens may lead to spurious results and data misinterpretation (Vaught and Lockhart 2012). This is true for tissues as well as for body fluids and the quality of these samples for further molecular analysis depends on the pre-analytical conditions in which they were acquired (Bernini et al. 2011; Cacciatore et al. 2013; David et al. 2014; Emwas et al. 2014; Juhl 2010). Furthermore, the lifestyle, and more important medication administered to a patient influence the molecular signatures in corresponding biological samples.

The human metabolome is composed by an ensemble of several thousands of small molecules (<1500–2000 Da) produced by the genome of the host organism and by the genomes of its microflora, or deriving from food, drinks, drugs or pollutants (Wishart et al. 2013), present on a very ample range of concentrations (from <1 nM to >1 μM). Blood plasma is a primary carrier of small molecules in the body. In terms of chemical composition, the blood plasma/serum metabolome is composed by hydrophobic (glycerides, phospholipids, fatty acids, steroids, fats, steroids) and hydrophilic (e.g. amino acids, glucose, glycerol, lactate, creatinine) molecules (Psychogios et al. 2011) whose relative concentrations reflect tissue lesions and organ dysfunctions. As a consequence, metabolomics of serum and plasma is increasingly used for successful patient stratification in various diseases including colorectal cancer (CRC), where both biofluids have shown to possess a strong signature of the disease that allows discrimination of CRC patients from healthy individuals and, in serum, permitted prediction of the overall survival within a set of metastatic patients (Bertini et al. 2012; Ma et al. 2012; Turano 2014; Zhang et al. 2014). The NMR detectable part of the serum/plasma metabolome generally consists of about 50 main compounds (Psychogios et al. 2011) and changes in the relative concentration of the molecules are at the bases of the metabolomic fingerprinting of various diseases. Effectively, the entire NMR spectral pattern becomes the relevant “biomarker” rather than a single or a few particular molecules.

The accuracy of metabolic profiles for clinical applications requires that the chemical composition (both in terms of nature and levels of detectable molecules) represents as much as possible the metabolome of the original sample, and therefore it is little affected by pre-analytical procedures. This aspect has received particular attention in recent years and it has led to the development of validated protocols for the collection/handling/storage of samples dedicated to future metabolomic applications (Bernini et al. 2011; Pinto et al. 2014; Vuckovic 2012; Yang et al. 2013). In particular, the factors influencing the stability of metabolites during the various steps of the pre-analytical phase for plasma and serum have been identified, leading to the development of ad hoc standard operating procedures concerning samples for metabolomics studies (Bernini et al. 2011). These procedures are beginning to be implemented in some biobanks. On the other hand, the possibility to use one sample for multiple in vitro approaches may offer advantages in studies aimed at combining different molecular profiles (genomics, transcriptomic, proteomics, and metabolomics).

Here, we have explored for the first time the possibility to extract a disease signature from NMR spectra of plasma obtained using Ficoll as separation medium and collected from patients with non-metastatic colorectal cancer (CRC) and metastatic colorectal cancer with liver-metastasis (LC) patients. Additionally, we have evaluated the impact of anesthesia on the metabolomic signature of the disease. This work contributes to the definition of some best practice pre-analytical procedures related to sample collection in NMR-based metabolomics studies. The observed changes that are specific for the different anesthetics within the same group of patients, on a long-term perspective may pave the way to the use of blood metabolomics for the development of personalized sedation procedures.

## Materials and methods

### Patients recruitment

This study was conducted in accordance with the ethical principles of Good Clinical Practice and the Declaration of Helsinki. The local Ethics Committee approved the protocol before commencement of the study, and all subjects gave written informed consent.

Seventy tumor patients, 32 female and 38 male, over 18 years of age, 40 with confirmed colorectal cancer and 30 with confirmed liver metastasis from colorectal cancer, were recruited for this study, as summarized in Table 1. All the participating subjects were able to understand the study design.

**Table 1:** Main demographic and clinical features of the patients’ cohort

	CRC patients	LC patients
Number	40	30
Hospital		
A	16	0
B	5	24
C	0	25
Sex		
Male	19	19
Female	21	11
Age		
Median	67.5	65
Range	44–89	42–78
Body mass index		
Underweight	1	2
Normal weight	20	18
Overweight	14	5
Obese	5	5
Stage		
0	1	0
I	8	0
IIA	9	0
IIC	1	0
IIIA	2	0
IIIB	5	0
IIIC	1	0
IV	1	29
IVA	8	0
IVB	2	0
n/a	2	1
Grading		
G2	25	11
G3	14	1
n/a	1	18
Tumor type		
Primary tumor	39	0
Second resection	1	0
Metastatsis	0	29
n/a	0	1
Anesthetic		
Etomidate	15	30
Propofol	24	0
Etomidate and Propofol	1	0

Included subjects with CRC had a tumor mass greater than 3 cm confirmed by colonoscopy and histology. Subjects with LC had liver metastasis greater than 3 cm confirmed by an imaging method (e.g. CT). For all the patients either hemihepatectomy or surgical tumor resection of rectal or sigmoid tumors were scheduled. Female patients of childbearing potential had to have a negative urine or serum pregnancy test.

Exclusion criteria used in the selection of the subjects included patients incapable of providing informed consent, patients treated with a neo-adjuvant treatment (chemotherapy or radiation) within the preceding 3 weeks, pregnant or lactating women, patients participating in another study of investigational drugs or devices parallel to, or less than 1 month before study entry, or previous participation in this study. In addition were excluded from the study employees or family members of the investigators.

### Blood collection and preparation

For each subject, blood was collected twice, the first time prior to the administration of the anesthesia and the second time when the patient was fully anesthetized, but before the surgery. All blood samples were drawn from the central infusion line and the blood was collected into commercially available EDTA-treated vials.

All vials were bandaged with an absorbent cloth, stored in a polythene bag and then immediately transferred at 4 °C. Plasma samples were obtained according to Indivumed standard operating procedures: EDTA-plasma was separated from the entire blood by a density gradient centrifugation using the Ficoll-Paque and a Leucosep Tube allowing also the separation of the mononuclear cells lymphocytes used for other aims. Plasma samples were stored at −80 °C until analysis.

### NMR sample preparation

Frozen plasma samples were thawed at room temperature and shaken before use. According to standard procedures (Bernini et al. 2011), a total of 300 µL of a phosphate sodium buffer (70 mM Na_2_HPO_4_; 20 % (v/v) ^2^H_2_O; 0.025 % (v/v) NaN_3_; 0.8 % (w/v) sodium trimethylsilyl [2,2,3,3-^2^H_4_]propionate (TSP) pH 7.4) was added to 300 µL of each plasma sample, and the mixture was homogenized by vortexing for 30 s. A total of 450 µL of this mixture was transferred into a 4.25 mm NMR tube (Bruker BioSpin srl) for analysis.

### NMR spectra acquisition

Monodimensional ^1^H NMR spectra for all samples were acquired using a Bruker 600 MHz metabolic profiler (Bruker BioSpin) operating at 600.13 MHz proton Larmor frequency and equipped with a 5 mm CPTCI ^1^H-^13^C-^31^P and ^2^H-decoupling cryoprobe including a z axis gradient coil, an automatic tuning-matching and an automatic sample changer. A BTO 2000 thermocouple served for temperature stabilization at the level of approximately 0.1 K at the sample. Before measurement, samples were kept for at least 3 min inside the NMR probe head, for temperature equilibration (300 K).

According to common practices (Beckonert et al. 2007; Gebregiworgis and Powers 2012; Padeletti et al. 2014; Pinto et al. 2015), for each sample, three monodimensional ^1^H NMR spectra were acquired with water peak suppression and different pulse sequences that allow the selective observation of different molecular components:

(i) a standard NOESY (Nuclear Overhauser Effect Spectroscopy) (Mckay 2011) 1Dpresat (noesygppr1d.comp; Bruker BioSpin) pulse sequence, using 64 scans, 98,304 data points, a spectral width of 18,028 Hz, an acquisition time of 2.7 s, a relaxation delay of 4 s and a mixing time of 0.1 s. This pulse sequence is designed to obtain a spectrum in which both signals of metabolites and high molecular weight molecules (lipids and lipoproteins) are visible.

(ii) a standard Carr-Purcell-Meiboom-Gill (CPMG) (Carr & Purcell 1954) (cpmgpr1d.comp; Bruker BioSpin) pulse sequence, using 64 scans, 73,728 data points, a spectral width of 12,019 Hz and a relaxation delay of 4 s. This pulse sequence is designed for the selective observation of small molecule components in solutions containing macromolecules.

(iii) a standard DIFFUSION-EDITED (Tang et al. 2004) (ledbgppr2s1d.comp; Bruker BioSpin) pulse sequence, using 64 scans, 98,304 data points, a spectral width of 18,028 Hz and a relaxation delay of 4 s. This pulse sequence is designed for the selective observation of macromolecule components in solutions containing small molecules; the resulting spectrum is generally made up only with the lipid, lipoprotein and protein signals.

Although the CPMG spin-echo was not sufficient to filter out the signals of the Ficoll polymeric components, the CPMG spectra resulted more informative than the other two types of spectra, providing the highest discrimination accuracies in the various multivariate comparisons performed. This finding indicates that the main differences among the analyzed groups arise from the small molecules profiles rather than from the macromolecular components.

### NMR spectra processing

**Fig. 1:**
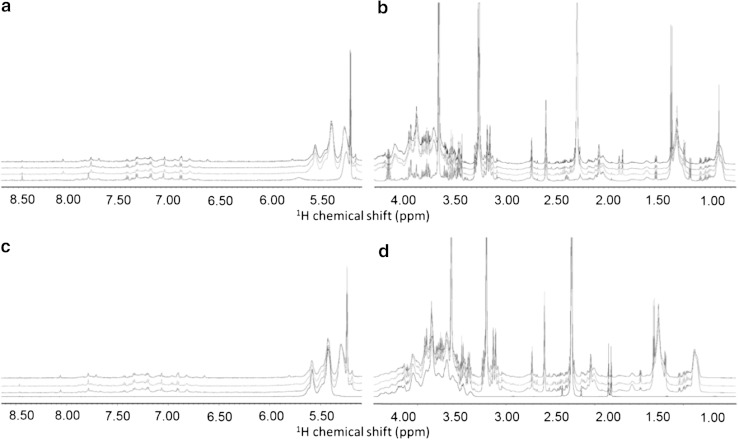
**a** Downfield (1.00–4.00 ppm) and **b** upfield (5.50–8.50 ppm) region of the ^1^H NMR CPMG spectra of plasma. The figure *underlines* the difference between a “normal” plasma spectrum (*red trace*) and the spectra of Ficoll-plasma samples of CRC patients (*green*, *blue* and *black traces*). The *baseline* is heavily distorted in the range 3.30–4.64 ppm and 5.20–5.65 ppm due to the presence of broad unresolved features. Comparison of c. downfield (1.00–4.00 ppm) and d. upfield (5.50–8.50 ppm) region of the ^1^H NMR CPMG spectra of the Ficoll-plasma samples of CRC patients (*blue*, *green* and *red traces*) and of Ficoll solution (*black trace*) (Color figure online)

Free induction decays were multiplied by an exponential function equivalent to a 1.0 Hz line-broadening factor before applying Fourier transform. Transformed spectra were automatically corrected for phase and baseline distortions and calibrated (glucose doublet at 5.24 ppm) using TopSpin 2.1 (Bruker Biospin srl). Each spectrum in the range between 0.20 and 10.00 ppm was segmented into 0.02-ppm chemical shift bins, and the corresponding spectral areas were integrated using the AMIX software (Bruker BioSpin). Regions between 6.00 and 3.30 ppm containing residual water signal and the broad and intense absorption attributable to the Ficoll contamination (Fig. 1 and Supplementary Figure S1) were excluded in subsequent statistical analyses. The total spectral area was calculated on the remaining bins and total area normalization was carried out on the data prior to pattern recognition.

### Statistical analysis

Various kinds of multivariate and univariate statistical techniques were applied on the obtained buckets using R 3.0.2 in house scripts. Principal component analysis (PCA) was used to obtain a preliminary outlook of the data (visualization in a reduced space, clusters detection, screening for outliers), partial least squares (PLS) and multilevel PLS (MPLS) were employed to perform supervised data reduction and classification. Canonical analysis (CA) was used in combination with PLS to increase the supervised separation of the analyzed groups. Accuracy, specificity and sensitivity were estimated according to standard definitions. The global accuracy for classification was assessed by means of a Monte carlo cross-validation scheme. Twenty metabolites (plus the antibiotic Cefuroxime) (Supplementary Table S2), whose peaks in the spectra were well defined and resolved, were assigned. Signal identification was performed using a library of NMR spectra of pure organic compounds, public databases (such as HMBD, human metabolic database, and SDBS, spectra database for organic compounds) storing reference NMR spectra of metabolites, spiking NMR experiments and literature data (Psychogios et al. 2011). The relative concentrations of the various metabolites in the different spectra were calculated by integrating the signal area (Wishart 2008). The Wilcoxon test was used for the determination of the meaningful metabolites. False discovery rate correction was applied using the Benjamini & Hochberg method (FDR) (Benjamini and Hochberg 2000): an adjusted *p* value of 0.05 was considered statistically significant. The changes in metabolites levels between two groups of spectra are calculated as the log_2_ fold change (FC) ratio of the normalized median intensities of the corresponding signals in the spectra of the two groups (Fold change). When two groups were constituted by individuals that could be compared in pairs (e.g. the same individual before and after anesthesia) a pairwise analysis was performed. Pairwise Wilcoxon test (univariate statistics) and multilevel PLS (van Velzen et al. 2008; Westerhuis et al. 2010) were used to this purpose.

### Data deposition

The NMR data are available from the MetaboLights database (www.ebi.ac.uk/metabolights) with the accession number MTBLS172.

## Results and discussion

### ^1^H NMR profiling of plasma separated by Ficoll

All the ^1^H NMR spectra of plasma separated by Ficoll were different from standard plasma spectra due to the presence of a broad and intense absorption from 3.30 to 4.64 ppm (Fig. 1 and Supplementary Figure S1). This absorption, together with sharper signals at 1.81, 1.84, 2.26, 5.42 and 5.58 ppm (Supplementary Figures S2 and S3), was attributed to contamination by the Ficoll used for plasma separation, as demonstrated by comparison with the ^1^H NMR CPMG spectrum acquired on a pure Ficoll solution (Fig. 1). Ficoll is a high molecular mass (400 kDa) hydrophilic branched polymer formed by copolymerization of sucrose with epichlorohydrin. CPMG pulse sequences, which are designed for the selective observation of small molecule components in solutions containing macromolecules, were not effective in removing the signals of Ficoll, most probably due to internal dynamics of the polymer that does not allow magnetization selection based on relaxation times. The Ficoll spectrum contains several signals that appear with different relative intensities in contaminated plasma spectra. This made essentially impossible a reliable subtraction of the Ficoll spectrum from those of plasma samples. The presence of this contaminant and in particular of the broad and intense enveloped centered at 3.80 ppm largely limited the applicability of standard procedures for spectral analysis, which are based on the comparison of spectra with comparable baselines. The analysis of the metabolomic profiles was therefore performed by systematically excluding the central part of the spectra (between 3.33 and 6.00 ppm, which includes the water signal). The use of a restricted active spectral area severely limited the number of molecules that might contribute to the spectral signature because the excluded spectral range contains signals form a large number of metabolites (including glucose, proline, serine, and threonine).

### Pre-anesthesia fingerprinting of CRC versus LC

**Fig. 2:**
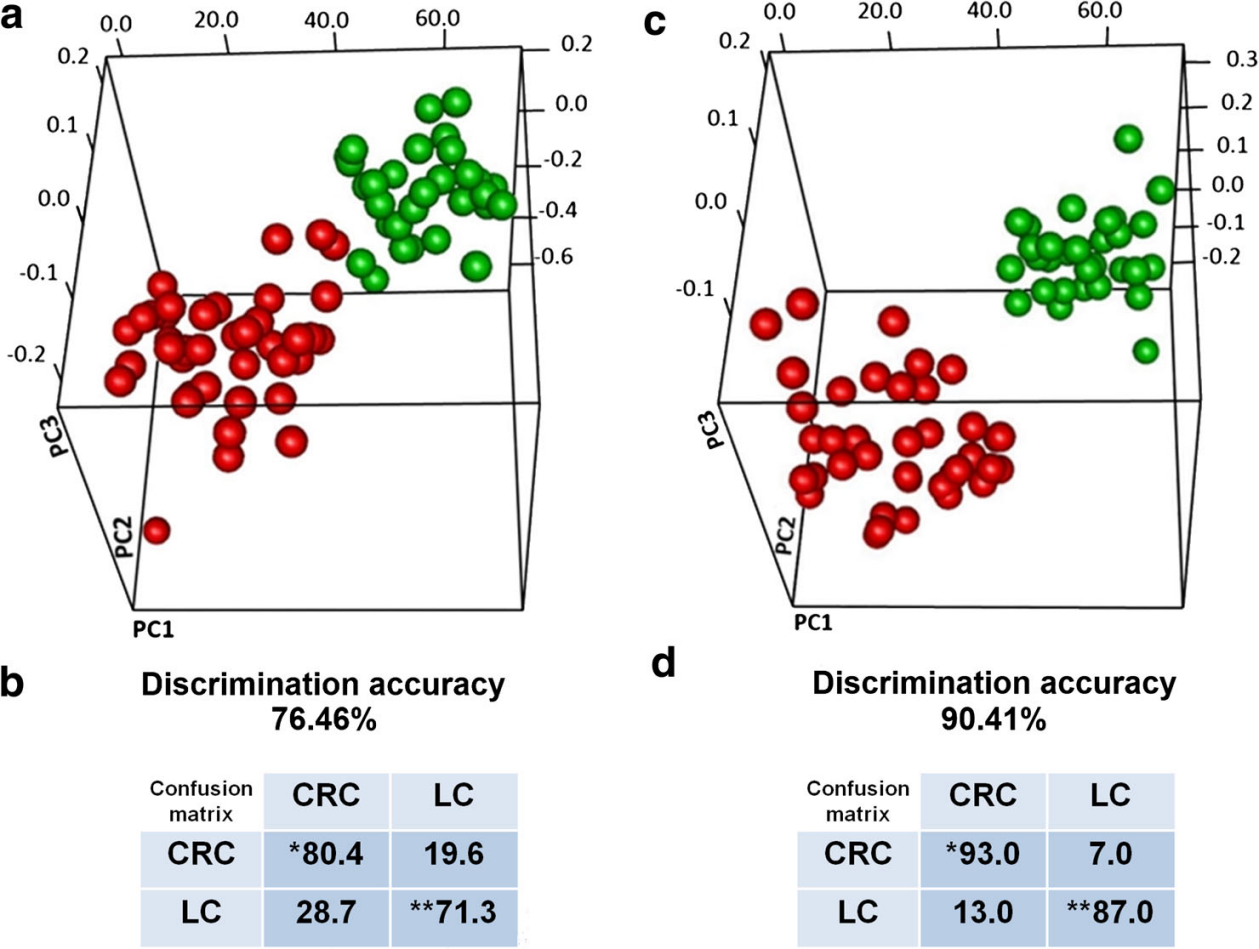
**a** Score plot of PLS-CA discrimination of CRC and LC patients using pre-anesthesia samples. *Red dots* samples from CRC patients. *Green dots* samples from LC patients. The *plot* has been constructed using ^1^H NMR CPMG spectra. **b** Cross validation test and confusion matrix for CRC and LC in pre-anesthesia samples; * sensitivity of the test; ** specificity of the test. **c** Score plot of PLS-CA discrimination of CRC and LC patients using post-anesthesia samples. *Red dots* samples from CRC patients. *Green dots* samples from LC patients. The *plot* has been constructed using ^1^H NMR CPMG spectra. **d** Cross validation test and confusion matrix for CRC and LC in post-anesthesia samples; * sensitivity of the test; ** specificity of the test (Color figure online)

The comparison between the ^1^H NMR profiles CRC and LC plasma samples is potentially relevant for the definition of the metabolomic signature of the disease progression. A defined metabolomic signature of CRC has been detected in a number of studies based on the metabolic fingerprinting of plasma or serum samples of CRC patients with respect to those of healthy controls, reaching discrimination accuracies up to 93.5 % (Ma et al. 2012). This figure rose up to 100 % when comparing healthy subjects and metastatic patients (Bertini et al. 2012). Here samples collected before anesthesia were analyzed to avoid the confounding effects deriving from the use of different sedation procedures. The samples were divided into two groups: the first contained all the pre-anesthesia of CRC patients; the second group contained all the pre-anesthesia samples belonging to LC patients. The discrimination between LC and CRC obtained from the comparison of the spectral active area of the corresponding plasma samples was relatively weak (Fig. 2a and Supplementary Figure S4), with discrimination accuracies ranging from 74 to 76 %, depending on the type of ^1^H NMR spectrum (NOESY = 74.84 %, CPMG = 76.46 %, Diffusion-edited = 73.80 %) employed for the analyses (Fig. 2b). The value of discrimination accuracy represents the predictability of the model, i.e. the percentage of correct classification testing the model with an unknown sample. A percentage value of 76 % is quite low to allow the use of this model in clinic analysis. Certainly this low value depend on the restricted spectral areas used due to Ficoll contamination, excluding from the analysis some metabolite peaks that might contributed to the spectral signature of CRC and LC samples.

**Fig. 3:**
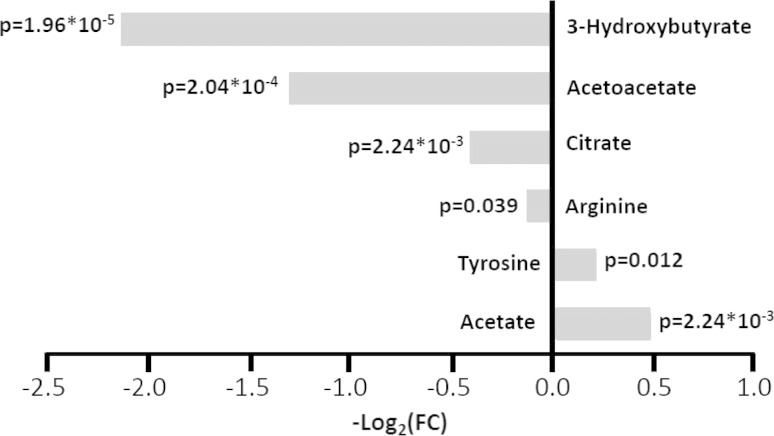
Metabolites whose concentrations are significantly different (*p* value < 0.05) in pre-anesthesia plasma samples from CRC patients compared to LC patients. The values of −Log_2_ (FC) and the *p* values are provided. Metabolites with −Log_2_ (FC) negative values have significantly higher concentration in plasma samples from CRC patients with respect to LC patients. Metabolites with −Log_2_ (FC) positive values have lower concentration in plasma samples from CRC patients with respect to LC patients

Still, statistically significant differences in the concentration of some metabolites were identified, as summarized in Fig. 3. Most of these metabolites had been previously identified as characteristic (up- or down-regulated in serum/plasma of CRC and metastatic CRC patients with respect to healthy subjects (Bertini et al. 2012; Ikeda et al. 2012; Leichtle et al. 2012; Ma et al. 2009; Miyagi et al. 2011; Nishiumi et al. 2012; Tan et al. 2013).

### Post-anesthesia fingerprinting of CRC versus LC

The discrimination between CRC and LC patients collected after anesthesia was performed by dividing the spectra into two groups: the first contained all post-anesthesia plasma samples from CRC patients; the second group contained all post-anesthesia plasma samples from LC. The discrimination accuracy between the two groups ranged from 85 to 90 % based on the type of spectrum (NOESY = 90.35 %, CPMG = 90.41 %, Diffusion-edited = 85.48 %) that we considered (Fig. 2c, d and Supplementary Figure S5). These data indicate that after anesthesia the two groups are well discriminated from each other. The increased discrimination, however, is attributable to the different pharmaceutical treatments administered to patients with colorectal cancer with respect to those with liver metastasis, rather than to the metabolomic signature of the disease. In general, the sedation procedures included treatment with different anesthetics (etomidate, propofol 1 %, propofol 2 %), contemporary administration of different antibiotics (cefuroxime, metronidazol), physiological solutions of different saline composition (ringer, ringer acetate, ringer lactate) and other drugs, as summarized in Supplementary Table S1.

The definition of best pre-analytical practices for accurate results of metabolomics analysis is an active field of research (Bernini et al. 2011; Fernandez-Peralbo and Luque de Castro 2012; Fliniaux et al. 2011; Pinto et al. 2014; Vuckovic 2012; Yang et al. 2013) and the impact of common clinical procedures and medical intervention have started to be considered (Cacciatore et al. 2013). The present results clearly indicated that the NMR metabolic profiles of samples collected immediately after drug administration contained a strong signature of the pharmaceutical treatment that might obscure the disease signature. Only a careful annotation of the administered molecules allows interpreting the changes occurring in the metabolome composition and permits spectral assignment.

### Effects of anesthesia on the metabolomic profiles

**Fig. 4:**
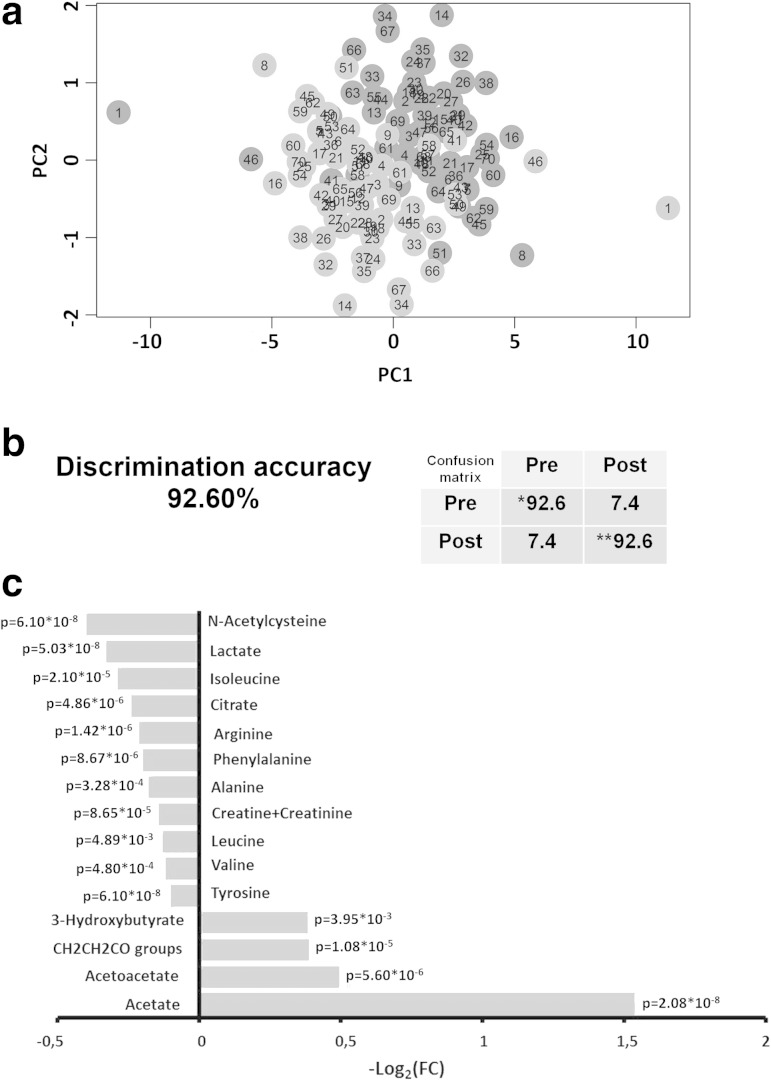
**a** Score plot of Multilevel PLS discrimination of pre- and post-anesthesia CRC and LC samples. *Red dots* pre-anesthesia samples from CRC and LC patients. *Green dots* post-anesthesia samples from CRC and LC patients. The *plot* has been constructed using ^1^H NMR CPMG spectra. In this type of informed analysis samples from the same donor are compared pairwise. **b** Cross validation pairwise test and confusion matrix for pre-anesthesia CRC and LC samples (PRE) and post-anesthesia CRC and LC samples (POST); * sensitivity of the test; ** specificity of the test. Metabolites whose concentration is significantly different (*p* value < 0.05) in pre-anesthesia plasma samples from CRC and LC patients with respect to post-anesthesia samples. The values of −Log_2_ (FC) and the p-values are provided. Metabolites with −Log_2_(FC) negative values have significantly lower concentration in post-anesthesia plasma samples from CRC and LC patients with respect to the pre-anesthesia samples. Metabolites with −Log_2_ (FC) positive values have higher concentration in post-anesthesia plasma samples from CRC and LC patients with respect to the pre-anesthesia samples (Color figure online)

The evaluation of the overall metabolic effect of the anesthesia on all plasma samples was performed using all sample divided into two groups: the first contained all the samples collected before anesthesia, independently on the cancer type; the second group contained all the samples collected after anesthesia. In this case, the MPLS approach was used to characterize the within-subject changes introduced in the individual metabolic profile by anesthesia. With this approach, the between-subject variations are removed and only the within-subject treatment-related variations are considered. The two groups are well discriminated (Fig. 4a and Supplementary Figure S6). The discrimination accuracy ranged from 90 to 93 % (Fig. 4b), depending on the type of spectrum (NOESY = 92.43 %, CPMG = 92.57 %, Diffusion-edited = 90.43 %). Some peaks, which turned out to be the major discriminants in the analyzed spectra areas, have been assigned to drugs administered to patients. This is the case of the antibiotic Cefuroxime, whose characteristic peaks are detectable only in spectra of patients treated with this molecule.

Additionally, the concentration of the metabolites whose peaks are assigned to well-known low molecular weight components of plasma have been evaluated to determine changes in their concentration after the anesthesia. The analysis was performed in the CPMG spectra. The Fig. 4c lists the metabolites whose concentration is significantly (*p* value < 0.05) decreased or increased in post-anesthesia plasma samples. A general decrease in metabolites concentration was observed and it can be reasonably attributed to a slower metabolism under anesthesia. The increased levels of a few molecules are more difficult to interpret but they might be directly related to the effect of some of the medical treatments, for example acetate concentration could increase because of the administration of Ringer Acetate solution (Supplementary Table S1).

**Fig. 5:**
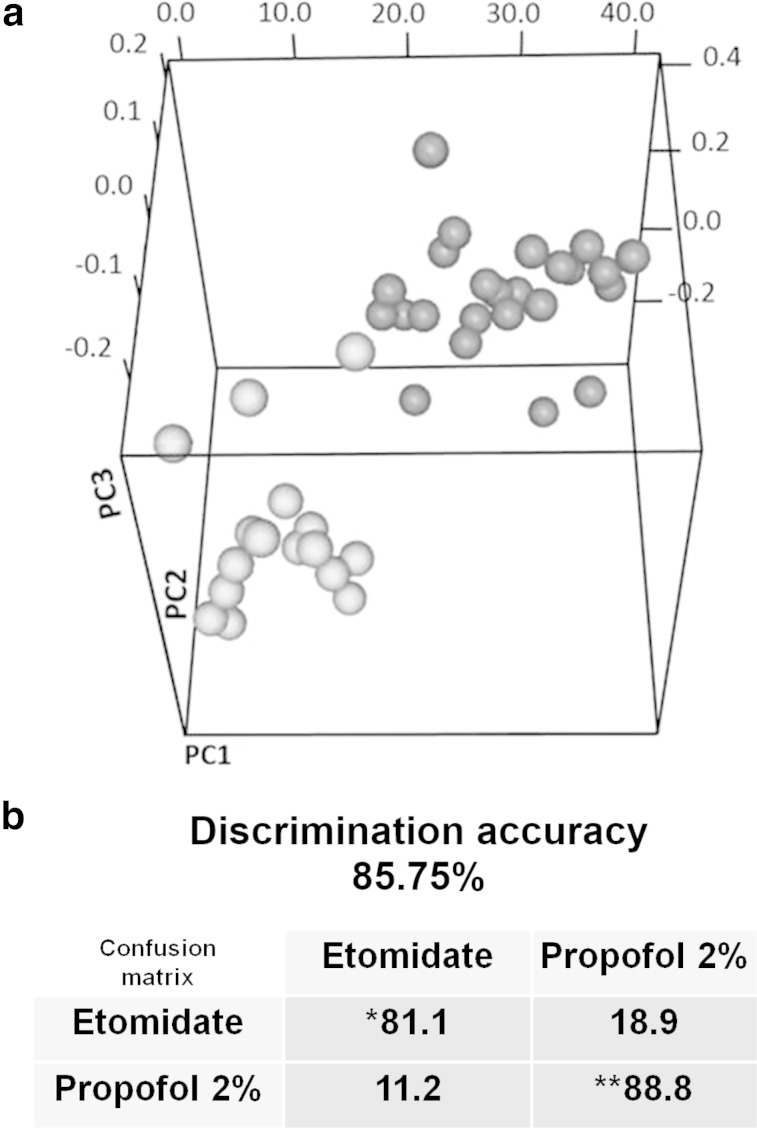
**a** Score plot of PLS-CA discrimination of post-anesthesia CRC samples from patients treated with different anesthetics. *Cyan dots* samples from CRC patients treated with etomidate. *Magenta dots* samples from CRC patients treated with propofol 2 %. The plot has been constructed using ^1^H NMR CPMG spectra. **b** Cross validation pairwise test and confusion matrix for plasma samples from CRC patients treated with different anesthetics;* sensitivity of the test; ** specificity of the test

We further analyzed the overall effect of anesthesia on the metabolic profiles focusing on a homogeneous set of patients, namely the CRC patients. Within this group, two different types of sedations were used: Etomidate and Propofol 2 %. Samples were divided into two groups: the first contained the samples collected after anesthesia belonging to CRC patients treated with Etomidate; the second group contained the samples collected after anesthesia belonging to CRC patients treated with Propofol 2 %. The discrimination accuracy was relatively high, of the order of 86 % (Fig. 5a, b and Supplementary Figure S7). The main molecular markers for the observed differences were branched amino acids such as isoleucine (−Log_2_ (FC) = 0.234), leucine (−Log_2_ (FC) = 0.349) and valine (−Log_2_ (FC) = 0.06). Their levels were less reduced under Propofol 2 % than under Etomidate sedation, although the measured changes are not statistically significant.

## Concluding remarks

An incredible amount of human biosamples currently stored in biobanks have been collected and processed by methods that did not anticipate the demands of metabolomic technologies; these samples represent a precious resource for metabolomics research, especially when focused on retrospective studies. Here we evaluated the reliability of NMR-based metabolomics data on plasma obtained from well-established lymphocyte separation via Ficoll in 2010, when metabolomics was not part of the design study. Although, this lymphocyte separation media is widely used and commonly applied to blood samples, we have shown that this procedure for plasma separation severely limits the NMR spectral range amenable for metabolomics analysis, excluding a number of molecules that contribute significantly to the NMR profile. Still, the active spectral area allowed us to measure meaningful differences between different groups of patients and it permitted the evaluation of the effect of anesthesia.

The changes occurring in the individual metabolome at the systemic levels during general anesthesia were monitored and some common trends observed, thus contributing another piece to the puzzle of the possible effects on the metabolome induced by medical intervention. From a methodological point of view, anesthesia introduces two confounding effects: (i) the signals of several molecules administered to the patients at the moment of the anesthesia are visible in the spectra, complicating the analysis of the NMR profiles; (ii) the levels of most plasma metabolites are decreased at variable degrees, thus altering the absolute and relative concentrations of metabolites between different groups of patients and reducing the discrimination capability of the method. Consequently, post anesthesia samples are not very suitable for standard metabolomics studies. Therefore, metabolomics studies aiming at the stratification of patients should be carefully conducted and patient follow-up data on medication is essential for the interpretation of metabolomic analyses.

The comparison of pre- and post-anesthesia plasma profiles, however, could provide hints at the molecular level of the consequences of general anesthesia. Prior work has shown in vivo cerebral metabolomic spectral patterns that are anesthetic-dependent (Jacob et al. 2012; Makaryus et al. 2011). Here we demonstrated that Propofol 2 % and Etomidate, which are known to act by selectively potentiating GABAa (γ-aminobutyric acid type A) receptors to provide very similar sedation, but to have some different side effects (Hemmings et al. 2005; Miner et al. 2007; Yip et al. 2013), induce small but meaningful differences in the plasma metabolic profiles at the systemic level. This molecular information is readily accessible via NMR profiling and may complement classical clinical signs for the evaluation of sedation agents and procedures.

## Electronic supplementary material

Below is the link to the electronic supplementary material.

(DOCX 2.06 Mb)

## Abbreviations

CA: Cano nical analysis
CPMG: Carr-purcell-meiboom-gill
CRC: Colorectal cancer
FDR: False discovery rate
GABAa: Γ-Aminobutyric acid type A
LC: Liver metastasis of colorectal cancer
MPLS: Multilevel PLS
MS: Mass spectrometry
NMR: Nuclear magnetic resonance
NOESY: Nuclear overhauser effect spectroscopy
PCA: Principal component analysis
PLS: Partial least squares
