# Supplementary material for: Metabolomics profiling of pre-and post-anesthesia plasma samples of colorectal patients obtained via Ficoll separation
Source: Metabolomics. 2015 Jul 25;11(6):1769–78. doi: 10.1007/s11306-015-0832-5 (PMC4605980; doi:10.1007/s11306-015-0832-5)
Supplement: Supplementary file 1 — (DOCX 2.06 Mb) [file 11306_2015_832_MOESM1_ESM.docx]

**SUPPLEMENTARY MATERIAL**

**Supplementary Tables**

**Supplementary Table S1** List of drugs administered to each patient concomitantly to anesthesia. Each patient is identified by a 3-characters alphanumeric code (NC, colorectal cancer; NL, liver metastasis of colorectal cancer).

| Patient | Anesthesia drugs | Dose | Unit |
| --- | --- | --- | --- |
| NC2 | sedat.&analg.: Ximovan | 7.5 | mg |
|  | sedat.&analg.: Dormicum | 7.5 | mg |
|  | antibiosis: Cefuroxime | 1500 | mg |
|  | loc.anesth.: Scandicain | 80 | mg |
|  | infus.&blood: Ringer solution | 3000 | ml |
|  | cardio: Akrinor | 100 | mg |
|  | opioid: Sufenta mite | 0.02 | mg |
|  | sedat.&analg.: Etomidate | 20 | mg |
|  | musc.relax.: Esmeron / Rocuronium | 30 | mg |
|  | antibiosis: Metronidazole / Clont | 500 | mg |
|  | inhal.anesth.: Desflurane | 12 | % |
|  | infus.&blood: Voluven | 1000 | ml |
|  | cardio: Akrinor | 100 | mg |
|  | inhal.anesth.: Desflurane | 10 | % |
|  | inhal.anesth.: Desflurane | 8 | % |
|  | inhal.anesth.: Desflurane | 6 | % |
|  | loc.anesth.: Carbostesin / Bubivacaine / Bucain | 25 | mg |
|  | inhal.anesth.: Desflurane | 8 | % |
|  | loc.anesth.: Carbostesin / Bubivacaine / Bucain | 15 | mg/h |
|  | musc.relax.: Esmeron / Rocuronium | 10 | mg |
|  | inhal.anesth.: Desflurane | 6 | % |
|  | musc.relax.: Esmeron / Rocuronium | 10 | mg |
|  | loc.anesth.: Carbostesin / Bubivacaine / Bucain | 10 | mg/h |
|  | infus.&blood: Packed red blood cells | 1 | n/a |
|  | infus.&blood: Fresh Frozen Plasma (FFP) | 1 | n/a |
|  | inhal.anesth.: Desflurane | 4 | % |
|  | infus.&blood: Fresh Frozen Plasma (FFP) | 1 | n/a |
| NC3 | sedat.&analg.: Dormicum | 7.5 | mg |
|  | infus.&blood: Ringer solution | 1000 | ml |
|  | sedat.&analg.: Etomidate | 20 | mg |
|  | opioid: Sufenta mite | 0.025 | mg |
|  | musc.relax.: Esmeron / Rocuronium | 40 | mg |
|  | loc.anesth.: Scandicain | 80 | mg |
|  | antibiosis: Metronidazole / Clont | 500 | mg |
|  | antibiosis: Cefuroxime | 1500 | mg |
|  | loc.anesth.: Sufenta | 0.025 | mg |
|  | cardio: Akrinor | 100 | mg |
|  | inhal.anesth.: Desflurane | 12 | % |
|  | inhal.anesth.: Desflurane | 6 | % |
|  | infus.&blood: Voluven | 500 | ml |
|  | inhal.anesth.: Desflurane | 8 | % |
|  | loc.anesth.: Carbostesin / Bubivacaine / Bucain | 15 | mg/h |
|  | diuretic&corticost.: Furosemide / Lasix | 5 | mg |
|  | diuretic&corticost.: Dexamethasone / Fortecortin | 4 | mg |
|  | cardio: Akrinor | 50 | mg |
|  | gastrointest.&antihist: Zofran | 4 | mg |
| NC4 | sedat.&analg.: Ximovan | 7.5 | mg |
|  | sedat.&analg.: Dormicum | 7.5 | mg |
|  | opioid: Sufenta mite | 0.025 | mg |
|  | sedat.&analg.: Etomidate | 20 | mg |
|  | infus.&blood: Ringer solution | 2000 | ml |
|  | antibiosis: Metronidazole / Clont | 500 | mg |
|  | inhal.anesth.: Desflurane | 6 | % |
|  | musc.relax.: Esmeron / Rocuronium | 40 | mg |
|  | loc.anesth.: Sufenta | 0.025 | mg |
|  | antibiosis: Cefuroxime | 1500 | mg |
|  | loc.anesth.: Carbostesin / Bubivacaine / Bucain | 30 | mg/h |
|  | cardio: Akrinor | 100 | mg |
|  | cardio: Akrinor | 100 | mg |
|  | musc.relax.: Esmeron / Rocuronium | 10 | mg |
|  | opioid: Sufenta mite | 0.01 | mg |
|  | infus.&blood: Voluven | 500 | ml |
|  | gastrointest.&antihist.: Zantic | 50 | mg |
|  | musc.relax.: Esmeron / Rocuronium | 10 | mg |
|  | cardio: Akrinor | 50 | mg |
|  | opioid: Sufenta mite | 0.01 | mg |
|  | inhal.anesth.: Desflurane | 7 | % |
| NC5 | sedat.&analg.: Dormicum | 7.5 | mg |
|  | infus.&blood: Ringer solution | 2000 | ml |
|  | loc.anesth.: Carbostesin / Bubivacaine / Bucain | 25 | mg |
|  | loc.anesth.: Scandicain | 80 | mg |
|  | cardio: Atropine | 0.5 | mg |
|  | musc.relax.: Atracurium / Tracrium | 40 | mg |
|  | sedat.&analg.: Etomidate | 20 | mg |
|  | opioid: Sufenta mite | 0.025 | mg |
|  | antibiosis: Metronidazole / Clont | 500 | mg |
|  | inhal.anesth.: Desflurane | 12 | % |
|  | antibiosis: Cefuroxime | 1500 | mg |
|  | inhal.anesth.: Desflurane | 8 | % |
|  | loc.anesth.: Sufenta | 0.025 | mg |
|  | inhal.anesth.: Desflurane | 6 | % |
|  | infus.&blood: Voluven | 500 | ml |
|  | gastrointest.&antihist.: Zantic | 50 | mg |
|  | cardio: Akrinor | 100 | mg |
|  | loc.anesth.: Carbostesin / Bubivacaine / Bucain | 15 | mg/h |
|  | musc.relax.: Atracurium / Tracrium | 10 | mg |
|  | inhal.anesth.: Desflurane | 8 | % |
| NC7 | sedat.&analg.: Ximovan | 7.5 | mg |
|  | sedat.&analg.: Dormicum | 7.5 | mg |
|  | infus.&blood: Ringer solution | 3000 | ml |
|  | sedat.&analg.: Etomidate | 20 | mg |
|  | musc.relax.: Atracurium / Tracrium | 40 | mg |
|  | opioid: Sufenta mite | 0.02 | mg |
|  | cardio: Akrinor | 100 | mg |
|  | inhal.anesth.: Sevofluran / Sevorane | 4 | % |
|  | antibiosis: Cefuroxime | 1500 | mg |
|  | inhal.anesth.: Sevofluran / Sevorane | 3 | % |
|  | antibiosis: Metronidazole / Clont | 500 | mg |
|  | cardio: Akrinor | 100 | mg |
|  | inhal.anesth.: Sevofluran / Sevorane | 4 | % |
|  | musc.relax.: Atracurium / Tracrium | 10 | mg |
|  | cardio: Akrinor | 50 | mg |
|  | musc.relax.: Atracurium / Tracrium | 10 | mg |
|  | infus.&blood: Expafusin | 500 | ml |
|  | inhal.anesth.: Sevofluran / Sevorane | 3.5 | % |
|  | opioid: Sufenta mite | 0.005 | mg |
|  | musc.relax.: Atracurium / Tracrium | 10 | mg |
|  | musc.relax.: Atracurium / Tracrium | 25 | mg |
|  | sedat.&analg.: Propofol 2 % | 400 | mg |
|  | opioid: Remifentanil / Ultivaperfusor | 0.8 | mg/h |
|  | cardio: Akrinor | 25 | mg |
|  | opioid: Remifentanil / Ultivaperfusor | 0.64 | mg/h |
|  | sedat.&analg.: Propofol 2 % | 300 | mg |
|  | cardio: Akrinor | 50 | mg |
|  | infus.&blood: Packed red blood cells | 2 | n/a |
| NC8 | sedat.&analg.: Ximovan | 7.5 | mg |
|  | sedat.&analg.: Dormicum | 7.5 | mg |
|  | infus.&blood: Ringer solution | 2000 | ml |
|  | cardio: Atropine | 0.5 | mg |
|  | opioid: Sufenta mite | 0.015 | mg |
|  | antibiosis: Cefuroxime | 1500 | mg |
|  | antibiosis: Metronidazole / Clont | 500 | mg |
|  | sedat.&analg.: Etomidate | 20 | mg |
|  | musc.relax.: Atracurium / Tracrium | 30 | mg |
|  | musc.relax.: Atracurium / Tracrium | 10 | mg |
|  | cardio: Akrinor | 50 | mg |
|  | loc.anesth.: Carbostesin / Bubivacaine / Bucain | 10 | mg/h |
|  | infus.&blood: Hydroxyethyl starch | 500 | ml |
|  | cardio: Akrinor | 50 | mg |
|  | sedat.&analg.: Perfalgan / Paracetamol | 1000 | mg |
|  | opioid: Sufenta mite | 0.005 | mg |
|  | musc.relax.: Atracurium / Tracrium | 10 | mg |
|  | opioid: Sufenta mite | 0.005 | mg |
| NC9 | infus.&blood: Ringer solution | 3000 | ml |
|  | antibiosis: Metronidazole / Clont | 500 | mg |
|  | sedat.&analg.: Etomidate | 14 | mg |
|  | antibiosis: Cefuroxime | 1500 | mg |
|  | musc.relax.: Atracurium / Tracrium | 20 | mg |
|  | opioid: Sufenta mite | 0.015 | mg |
|  | inhal.anesth.: Desflurane | 8 | % |
|  | sedat.&analg.: Etomidate | 4 | mg |
|  | inhal.anesth.: Desflurane | 6 | % |
|  | cardio: Akrinor | 70 | mg |
|  | musc.relax.: Atracurium / Tracrium | 10 | mg |
|  | opioid: Sufenta mite | 0.01 | mg |
|  | inhal.anesth.: Desflurane | 8 | % |
|  | diuretic&corticost.: Dexamethasone / Fortecortin | 4 | mg |
|  | gastrointest.&antihist: Zofran | 4 | mg |
|  | musc.relax.: Atracurium / Tracrium | 10 | mg |
| NC10 | sedat.&analg.: Ximovan | 7.5 | mg |
|  | infus.&blood: Ringer solution | 2000 | ml |
|  | antibiosis: Cefuroxime | 1500 | mg |
|  | sedat.&analg.: Etomidate | 20 | mg |
|  | opioid: Sufenta mite | 0.02 | mg |
|  | musc.relax.: Esmeron / Rocuronium | 40 | mg |
|  | cardio: Atropine | 0.25 | mg |
|  | inhal.anesth.: Sevofluran / Sevorane | 2.5 | % |
|  | antibiosis: Metronidazole / Clont | 500 | mg |
|  | inhal.anesth.: Sevofluran / Sevorane | 2 | % |
|  | opioid: Sufenta mite | 0.005 | mg |
|  | inhal.anesth.: Sevofluran / Sevorane | 1.8 | % |
|  | cardio: Akrinor | 50 | mg |
| NC11 | sedat.&analg.: Ximovan | 7.5 | mg |
|  | sedat.&analg.: Dormicum | 7.5 | mg |
|  | antibiosis: Metronidazole / Clont | 500 | mg |
|  | infus.&blood: Ringer solution | 2000 | ml |
|  | antibiosis: Cefuroxime | 1500 | mg |
|  | loc.anesth.: Scandicain | 80 | mg |
|  | musc.relax.: Esmeron / Rocuronium | 50 | mg |
|  | opioid: Sufenta mite | 0.02 | mg |
|  | sedat.&analg.: Etomidate | 20 | mg |
|  | inhal.anesth.: Sevofluran / Sevorane | 2 | % |
|  | loc.anesth.: Carbostesin / Bubivacaine / Bucain | 25 | mg |
|  | musc.relax.: Atracurium / Tracrium | 25 | mg |
|  | musc.relax.: Atracurium / Tracrium | 25 | mg |
|  | loc.anesth.: Carbostesin / Bubivacaine / Bucain | 15 | mg/h |
|  | opioid: Sufenta mite | 0.01 | mg |
|  | musc.relax.: Atracurium / Tracrium | 25 | mg |
| NC12 | sedat.&analg.: Dormicum | 7.5 | mg |
|  | infus.&blood: Ringer solution | 2000 | ml |
|  | sedat.&analg.: Etomidate | 20 | mg |
|  | opioid: Sufenta mite | 0.025 | mg |
|  | musc.relax.: Esmeron / Rocuronium | 35 | mg |
|  | antibiosis: Cefuroxime | 1500 | mg |
|  | antibiosis: Metronidazole / Clont | 500 | mg |
|  | inhal.anesth.: Desflurane | 12 | % |
|  | inhal.anesth.: Desflurane | 10 | % |
|  | inhal.anesth.: Desflurane | 8 | % |
|  | inhal.anesth.: Desflurane | 7.5 | % |
|  | musc.relax.: Esmeron / Rocuronium | 10 | mg |
|  | infus.&blood: Hydroxyethyl starch | 500 | ml |
|  | opioid: Sufenta mite | 0.01 | mg |
|  | cardio: Akrinor | 50 | mg |
|  | musc.relax.: Esmeron / Rocuronium | 10 | mg |
|  | opioid: Sufenta mite | 0.015 | mg |
| NC13 | sedat.&analg.: Ximovan | 7.5 | mg |
|  | sedat.&analg.: Dormicum | 7.5 | mg |
|  | infus.&blood: Ringer solution | 2000 | ml |
|  | opioid: Sufenta mite | 0.025 | mg |
|  | sedat.&analg.: Etomidate | 20 | mg |
|  | inhal.anesth.: Desflurane | 18 | % |
|  | musc.relax.: Esmeron / Rocuronium | 40 | mg |
|  | inhal.anesth.: Desflurane | 12 | % |
|  | cardio: Clonidine / Catapresan | 0.1 | mg |
|  | antibiosis: Metronidazole / Clont | 500 | mg |
|  | inhal.anesth.: Desflurane | 8 | % |
|  | antibiosis: Cefuroxime | 1500 | mg |
|  | musc.relax.: Esmeron / Rocuronium | 10 | mg |
|  | opioid: Sufenta mite | 0.01 | mg |
|  | musc.relax.: Esmeron / Rocuronium | 10 | mg |
|  | musc.relax.: Esmeron / Rocuronium | 5 | mg |
| NC15 | sedat.&analg.: Dormicum | 7.5 | mg |
|  | infus.&blood: Ringer solution | 3000 | ml |
|  | loc.anesth.: Scandicain | 80 | mg |
|  | sedat.&analg.: Etomidate | 20 | mg |
|  | opioid: Sufenta mite | 0.02 | mg |
|  | cardio: Akrinor | 200 | mg |
|  | musc.relax.: Atracurium / Tracrium | 40 | mg |
|  | inhal.anesth.: Sevofluran / Sevorane | 3 | % |
|  | antibiosis: Cefuroxime | 1500 | mg |
|  | loc.anesth.: Carbostesin / Bubivacaine / Bucain | 10 | mg |
|  | cardio: Atropine | 0.25 | mg |
|  | cardio: Arterenolperfusor | 0.06 | mg/h |
|  | cardio: Akrinor | 100 | mg |
|  | antibiosis: Metronidazole / Clont | 500 | mg |
|  | opioid: Sufenta mite | 0.005 | mg |
|  | loc.anesth.: Carbostesin / Bubivacaine / Bucain | 12.5 | mg |
|  | opioid: Sufenta mite | 0.005 | mg |
|  | cardio: Arterenolperfusor | 0.09 | mg/h |
|  | musc.relax.: Atracurium / Tracrium | 10 | mg |
|  | infus.&blood: Voluven | 500 | ml |
|  | inhal.anesth.: Sevofluran / Sevorane | 4 | % |
|  | cardio: Arterenolperfusor | 0.12 | mg/h |
|  | cardio: Atropine | 0.25 | mg |
|  | cardio: Arterenolperfusor | 0.06 | mg/h |
|  | cardio: Arterenolperfusor | 0.12 | mg/h |
|  | inhal.anesth.: Sevofluran / Sevorane | 3.5 | % |
| NC16 | sedat.&analg.: Ximovan | 7.5 | mg |
|  | sedat.&analg.: Dormicum | 7.5 | mg |
|  | infus.&blood: Ringer solution | 2000 | ml |
|  | opioid: Sufenta mite | 0.02 | mg |
|  | sedat.&analg.: Etomidate | 20 | mg |
|  | musc.relax.: Atracurium / Tracrium | 35 | mg |
|  | antibiosis: Cefuroxime | 1500 | mg |
|  | cardio: Atropine | 0.25 | mg |
|  | antibiosis: Metronidazole / Clont | 500 | mg |
|  | inhal.anesth.: Sevofluran / Sevorane | 3 | % |
|  | musc.relax.: Atracurium / Tracrium | 20 | mg |
|  | opioid: Sufenta mite | 0.01 | mg |
|  | cardio: Akrinor | 100 | mg |
|  | opioid: Sufenta mite | 0.01 | mg |
|  | cardio: Akrinor | 100 | mg |
|  | inhal.anesth.: Sevofluran / Sevorane | 4 | % |
|  | musc.relax.: Atracurium / Tracrium | 20 | mg |
|  | opioid: Sufenta mite | 0.01 | mg |
|  | infus.&blood: Hydroxyethyl starch | 500 | ml |
|  | inhal.anesth.: Sevofluran / Sevorane | 3 | % |
| NC17 | sedat.&analg.: Dormicum | 7.5 | mg |
|  | infus.&blood: Ringer solution | 3000 | ml |
|  | musc.relax.: Esmeron / Rocuronium | 40 | mg |
|  | opioid: Sufenta mite | 0.025 | mg |
|  | sedat.&analg.: Etomidate | 20 | mg |
|  | inhal.anesth.: Sevofluran / Sevorane | 4.5 | % |
|  | antibiosis: Cefuroxime | 1500 | mg |
|  | antibiosis: Metronidazole / Clont | 500 | mg |
|  | opioid: Sufenta mite | 0.01 | mg |
|  | cardio: Akrinor | 100 | mg |
|  | musc.relax.: Esmeron / Rocuronium | 10 | mg |
|  | inhal.anesth.: Sevofluran / Sevorane | 4 | % |
|  | musc.relax.: Esmeron / Rocuronium | 10 | mg |
|  | opioid: Sufenta mite | 0.01 | mg |
|  | musc.relax.: Esmeron / Rocuronium | 20 | mg |
|  | opioid: Sufenta mite | 0.005 | mg |
|  | musc.relax.: Esmeron / Rocuronium | 10 | mg |
|  | opioid: Sufenta mite | 0.01 | mg |
|  | musc.relax.: Esmeron / Rocuronium | 10 | mg |
|  | opioid: Sufenta mite | 0.01 | mg |
|  | cardio: Akrinor | 50 | mg |
|  | sedat.&analg.: Perfalgan / Paracetamol | 1000 | mg |
|  | opioid: Sufenta mite | 0.01 | mg |
|  | infus.&blood: Voluven | 500 | ml |
| NC18 | sedat.&analg.: Dormicum | 7.5 | mg |
|  | infus.&blood: Ringer solution | 3000 | ml |
|  | musc.relax.: Pantolax / Lysthenon / Succinylcholine | 80 | mg |
|  | sedat.&analg.: Etomidate | 20 | mg |
|  | opioid: Sufenta mite | 0.02 | mg |
|  | sedat.&analg.: Etomidate | 10 | mg |
|  | antibiosis: Cefuroxime | 1500 | mg |
|  | inhal.anesth.: Sevofluran / Sevorane | 3 | % |
|  | antibiosis: Metronidazole / Clont | 500 | mg |
|  | musc.relax.: Atracurium / Tracrium | 30 | mg |
|  | cardio: Akrinor | 100 | mg |
|  | opioid: Sufenta mite | 0.01 | mg |
|  | cardio: Clonidine / Catapresan | 0.03 | mg |
|  | opioid: Sufenta mite | 0.005 | mg |
|  | opioid: Sufenta mite | 0.01 | mg |
|  | musc.relax.: Atracurium / Tracrium | 10 | mg |
|  | cardio: Clonidine / Catapresan | 0.03 | mg |
|  | infus.&blood: Voluven | 1000 | ml |
|  | musc.relax.: Atracurium / Tracrium | 10 | mg |
|  | musc.relax.: Atracurium / Tracrium | 10 | mg |
|  | opioid: Sufenta mite | 0.005 | mg |
|  | cardio: Akrinor | 50 | mg |
|  | musc.relax.: Atracurium / Tracrium | 10 | mg |
|  | infus.&blood: Packed red blood cells | 1 | n/a |
|  | infus.&blood: Packed red blood cells | 1 | n/a |
|  | opioid: Sufenta mite | 0.005 | mg |
|  | cardio: Arterenolperfusor | 0.4 | mg/h |
|  | cardio: Akrinor | 100 | mg |
| NC20 | sedat.&analg.: Dormicum | 3.75 | mg |
|  | loc.anesth.: Scandicain | 80 | mg |
|  | sedat.&analg.: Etomidate | 30 | mg |
|  | musc.relax.: Esmeron / Rocuronium | 40 | mg |
|  | opioid: Sufenta mite | 0.025 | mg |
|  | inhal.anesth.: Sevofluran / Sevorane | 4 | % |
|  | antibiosis: Metronidazole / Clont | 500 | mg |
|  | antibiosis: Cefuroxime | 1500 | mg |
|  | infus.&blood: Ringer solution | 2000 | ml |
|  | musc.relax.: Esmeron / Rocuronium | 10 | mg |
|  | loc.anesth.: Carbostesin / Bubivacaine / Bucain | 25 | mg |
|  | musc.relax.: Esmeron / Rocuronium | 10 | mg |
|  | inhal.anesth.: Sevofluran / Sevorane | 3 | % |
|  | musc.relax.: Esmeron / Rocuronium | 5 | mg |
|  | loc.anesth.: Carbostesin / Bubivacaine / Bucain | 25 | mg |
|  | musc.relax.: Esmeron / Rocuronium | 10 | mg |
|  | musc.relax.: Esmeron / Rocuronium | 10 | mg |
| NC21 | sedat.&analg.: Dormicum | 7.5 | mg |
|  | loc.anesth.: Naropin | 22.5 | mg |
|  | loc.anesth.: Naropin | 30 | mg |
|  | loc.anesth.: Sufenta | 0.01 | mg |
|  | inhal.anesth.: Sevofluran / Sevorane | 0.8 | % |
|  | musc.relax.: Norcuron / Vecuronium | 6 | mg |
|  | sedat.&analg.: Propofol 2 % | 200 | mg |
|  | opioid: Sufenta mite | 0.01 | mg |
|  | antibiosis: Metronidazole / Clont | 500 | mg |
|  | infus.&blood: Hydroxyethyl starch | 1500 | ml |
|  | sedat.&analg.: Dormicum | 3 | mg |
|  | antibiosis: Basocef / Cefazolin / Elzogram | 2000 | mg |
|  | infus.&blood: Ringeracetat | 1000 | ml |
|  | infus.&blood: Potassium chloride 7.45% | 20 | mval |
|  | inhal.anesth.: Sevofluran / Sevorane | 2.5 | % |
|  | musc.relax.: Norcuron / Vecuronium | 2 | mg |
|  | cardio: Akrinor | 50 | mg |
|  | infus.&blood: Glucose 5% | 500 | ml |
|  | musc.relax.: Norcuron / Vecuronium | 2 | mg |
|  | opioid: Sufenta mite | 0.01 | mg |
|  | musc.relax.: Norcuron / Vecuronium | 2 | mg |
|  | inhal.anesth.: Sevofluran / Sevorane | 2 | % |
|  | loc.anesth.: Naropin | 30 | mg |
|  | loc.anesth.: Sufenta | 0.01 | mg |
|  | cardio: Akrinor | 50 | mg |
|  | cardio: Akrinor | 100 | mg |
| NC22 | sedat.&analg.: Tranxilium | 25 | mg |
|  | sedat.&analg.: Dormicum | 7.5 | mg |
|  | opioid: Sufenta mite | 0.02 | mg |
|  | infus.&blood: Ringer solution | 1500 | ml |
|  | inhal.anesth.: Sevofluran / Sevorane | 2 | % |
|  | musc.relax.: Norcuron / Vecuronium | 8 | mg |
|  | sedat.&analg.: Propofol 2 % | 200 | mg |
|  | loc.anesth.: Naropin | 15 | mg |
|  | loc.anesth.: Naropin | 37.5 | mg |
|  | musc.relax.: Norcuron / Vecuronium | 2 | mg |
|  | opioid: Sufenta mite | 0.01 | mg |
|  | cardio: Akrinor | 50 | mg |
|  | infus.&blood: Hydroxyethyl starch | 1500 | ml |
|  | cardio: Akrinor | 50 | mg |
|  | infus.&blood: Fresh Frozen Plasma (FFP) | 4 | n/a |
|  | musc.relax.: Norcuron / Vecuronium | 2 | mg |
|  | opioid: Sufenta mite | 0.005 | mg |
|  | infus.&blood: Packed red blood cells | 7 | n/a |
| NC23 | sedat.&analg.: Tranxilium | 25 | mg |
|  | sedat.&analg.: Dormicum | 7.5 | mg |
|  | infus.&blood: Ringer solution | 1000 | ml |
|  | sedat.&analg.: Propofol 2 % | 200 | mg |
|  | antibiosis: Metronidazole / Clont | 500 | mg |
|  | musc.relax.: Norcuron / Vecuronium | 8 | mg |
|  | opioid: Sufenta mite | 0.04 | mg |
|  | inhal.anesth.: Sevofluran / Sevorane | 3 | % |
|  | antibiosis: Basocef / Cefazolin / Elzogram | 2000 | mg |
|  | sedat.&analg.: Dormicum | 5 | mg |
|  | cardio: Akrinor | 50 | mg |
|  | infus.&blood: Hydroxyethyl starch | 1000 | ml |
|  | musc.relax.: Norcuron / Vecuronium | 2 | mg |
|  | opioid: Sufenta mite | 0.01 | mg |
|  | cardio: Akrinor | 50 | mg |
|  | opioid: Sufenta mite | 0.02 | mg |
|  | musc.relax.: Norcuron / Vecuronium | 2 | mg |
|  | opioid: Sufenta mite | 0.03 | mg |
|  | cardio: Clonidine / Catapresan | 0.15 | mg |
|  | opioid: Sufenta mite | 0.02 | mg |
| NC25 | sedat.&analg.: Tranxilium | 25 | mg |
|  | sedat.&analg.: Dormicum | 7.5 | mg |
|  | infus.&blood: Ringeracetat | 1500 | ml |
|  | infus.&blood: Potassium chloride 7.45% | 20 | mval |
|  | loc.anesth.: Scandicain | 30 | mg |
|  | antibiosis: Basocef / Cefazolin / Elzogram | 2000 | mg |
|  | opioid: Sufenta mite | 0.04 | mg |
|  | sedat.&analg.: Propofol 2 % | 200 | mg |
|  | antibiosis: Metronidazole / Clont | 500 | mg |
|  | musc.relax.: Norcuron / Vecuronium | 8 | mg |
|  | sedat.&analg.: Propofol 2 % | 50 | mg |
|  | infus.&blood: Hydroxyethyl starch | 1000 | ml |
|  | cardio: Akrinor | 50 | mg |
|  | sedat.&analg.: Propofolperfusor | 600 | mg/h |
|  | loc.anesth.: Naropin | 30 | mg |
|  | loc.anesth.: Sufenta | 0.01 | mg |
|  | musc.relax.: Norcuron / Vecuronium | 2 | mg |
|  | opioid: Sufenta mite | 0.01 | mg |
|  | diuretic&corticost.: Solu Decortin H / Prednisolone | 50 | mg |
|  | musc.relax.: Norcuron / Vecuronium | 5 | mg |
|  | cardio: Akrinor | 50 | mg |
|  | opioid: Sufenta mite | 0.03 | mg |
|  | sedat.&analg.: Dormicum | 3 | mg |
|  | opioid: Sufenta mite | 0.02 | mg |
|  | inhal.anesth.: Sevofluran / Sevorane | 1.1 | % |
|  | cardio: Akrinor | 50 | mg |
|  | cardio: Akrinor | 50 | mg |
|  | sedat.&analg.: Novamin / Novalgin | 2500 | mg |
|  | diuretic&corticost.: Solu Decortin H / Prednisolone | 50 | mg |
|  | inhal.anesth.: Sevofluran / Sevorane | 0.6 | % |
| NC26 | sedat.&analg.: Tranxilium | 25 | mg |
|  | sedat.&analg.: Dormicum | 7.5 | mg |
|  | infus.&blood: Ringeracetat | 1500 | ml |
|  | infus.&blood: Hydroxyethyl starch | 1000 | ml |
|  | musc.relax.: Norcuron / Vecuronium | 8 | mg |
|  | inhal.anesth.: Sevofluran / Sevorane | 0.5 | % |
|  | opioid: Sufenta mite | 0.025 | mg |
|  | sedat.&analg.: Propofol 2 % | 200 | mg |
|  | cardio: Akrinor | 50 | mg |
|  | inhal.anesth.: Sevofluran / Sevorane | 0.9 | % |
|  | infus.&blood: Potassium chloride 7.45% | 20 | mval |
|  | opioid: Sufenta mite | 0.025 | mg |
|  | infus.&blood: Packed red blood cells | 1 | n/a |
|  | infus.&blood: Fresh Frozen Plasma (FFP) | 4 | n/a |
|  | inhal.anesth.: Sevofluran / Sevorane | 1 | % |
|  | opioid: Sufenta mite | 0.025 | mg |
|  | musc.relax.: Norcuron / Vecuronium | 2 | mg |
|  | opioid: Sufenta mite | 0.015 | mg |
|  | cardio: Arterenolperfusor | 2 | mg/h |
|  | infus.&blood: Packed red blood cells | 5 | n/a |
|  | infus.&blood: Packed red blood cells | 1 | n/a |
|  | cardio: Suprarenin | 0.02 | mg |
|  | cardio: Suprareninperfusor | 5 | mg/h |
| NC27 | sedat.&analg.: Tranxilium | 25 | mg |
|  | sedat.&analg.: Dormicum | 7.5 | mg |
|  | loc.anesth.: Naropin | 22.5 | mg |
|  | infus.&blood: Ringeracetat | 1500 | ml |
|  | infus.&blood: Potassium chloride 7.45% | 20 | mval |
|  | sedat.&analg.: Propofol 2 % | 200 | mg |
|  | opioid: Sufenta mite | 0.02 | mg |
|  | musc.relax.: Norcuron / Vecuronium | 7 | mg |
|  | inhal.anesth.: Sevofluran / Sevorane | 0.6 | % |
|  | loc.anesth.: Sufenta | 0.02 | mg |
|  | inhal.anesth.: Sevofluran / Sevorane | 0.7 | % |
|  | cardio: Akrinor | 50 | mg |
|  | inhal.anesth.: Sevofluran / Sevorane | 0.8 | % |
|  | loc.anesth.: Naropin | 7.5 | mg |
|  | musc.relax.: Norcuron / Vecuronium | 3 | mg |
|  | loc.anesth.: Naropin | 7.5 | mg |
|  | loc.anesth.: Naropin | 11.25 | mg |
|  | inhal.anesth.: Sevofluran / Sevorane | 1.6 | % |
|  | cardio: Akrinor | 100 | mg |
|  | cardio: Akrinor | 50 | mg |
|  | infus.&blood: Potassium chloride 7.45% | 20 | mval |
|  | cardio: Akrinor | 50 | mg |
|  | loc.anesth.: Naropin | 11.25 | mg |
|  | inhal.anesth.: Sevofluran / Sevorane | 1.3 | % |
|  | musc.relax.: Norcuron / Vecuronium | 4 | mg |
|  | cardio: Akrinor | 100 | mg |
|  | infus.&blood: Voluven | 1000 | ml |
|  | cardio: Akrinor | 50 | mg |
|  | cardio: Akrinor | 50 | mg |
|  | inhal.anesth.: Sevofluran / Sevorane | 1.1 | % |
|  | opioid: Sufenta mite | 0.01 | mg |
| NC28 | sedat.&analg.: Tranxilium | 25 | mg |
|  | gastrointest.&antihist: Zofran | 8 | mg |
|  | sedat.&analg.: Dormicum | 7.5 | mg |
|  | opioid: Sufenta mite | 0.005 | mg |
|  | antibiosis: Basocef / Cefazolin / Elzogram | 2000 | mg |
|  | infus.&blood: Ringer solution | 2000 | ml |
|  | sedat.&analg.: Dormicum | 1 | mg |
|  | antibiosis: Metronidazole / Clont | 500 | mg |
|  | inhal.anesth.: Sevofluran / Sevorane | 1.9 | % |
|  | opioid: Sufenta mite | 0.02 | mg |
|  | musc.relax.: Nimbex | 8 | mg |
|  | sedat.&analg.: Propofol 2 % | 200 | mg |
|  | loc.anesth.: Sufenta | 0.01 | mg |
|  | loc.anesth.: Naropin | 37.5 | mg |
|  | opioid: Sufenta mite | 0.015 | mg |
|  | infus.&blood: Potassium chloride 7.45% | 20 | mval |
|  | musc.relax.: Nimbex | 2 | mg |
|  | opioid: Sufenta mite | 0.01 | mg |
|  | sedat.&analg.: Propofolperfusor | 340 | mg/h |
|  | cardio: Atropine | 0.25 | mg |
|  | musc.relax.: Nimbex | 2 | mg |
|  | cardio: Akrinor | 25 | mg |
|  | opioid: Sufenta mite | 0.02 | mg |
|  | musc.relax.: Nimbex | 2 | mg |
|  | loc.anesth.: Naropin | 37.5 | mg |
| NC29 | sedat.&analg.: Tranxilium | 25 | mg |
|  | sedat.&analg.: Dormicum | 7.5 | mg |
|  | gastrointest.&antihist: Zofran | 8 | mg |
|  | antibiosis: Basocef / Cefazolin / Elzogram | 2000 | mg |
|  | antibiosis: Metronidazole / Clont | 500 | mg |
|  | opioid: Sufenta mite | 0.025 | mg |
|  | sedat.&analg.: Propofol 2 % | 200 | mg |
|  | musc.relax.: Norcuron / Vecuronium | 7 | mg |
|  | infus.&blood: Ringeracetat | 1000 | ml |
|  | infus.&blood: Potassium chloride 7.45% | 20 | mval |
|  | infus.&blood: Hydroxyethyl starch | 500 | ml |
|  | sedat.&analg.: Propofolperfusor | 345 | mg/h |
|  | opioid: Sufenta mite | 0.01 | mg |
|  | musc.relax.: Norcuron / Vecuronium | 3 | mg |
|  | opioid: Sufenta mite | 0.015 | mg |
|  | sedat.&analg.: Propofolperfusor | 379.5 | mg/h |
|  | musc.relax.: Norcuron / Vecuronium | 2 | mg |
| NC30 | sedat.&analg.: Tranxilium | 10 | mg |
|  | sedat.&analg.: Dormicum | 3.75 | mg |
|  | opioid: Sufenta mite | 0.01 | mg |
|  | sedat.&analg.: Propofol 2 % | 150 | mg |
|  | musc.relax.: Norcuron / Vecuronium | 6 | mg |
|  | infus.&blood: Ringeracetat | 1500 | ml |
|  | sedat.&analg.: Dormicum | 3 | mg |
|  | infus.&blood: Potassium chloride 7.45% | 20 | mval |
|  | antibiosis: Metronidazole / Clont | 500 | mg |
|  | inhal.anesth.: Sevofluran / Sevorane | 6 | % |
|  | antibiosis: Basocef / Cefazolin / Elzogram | 2000 | mg |
|  | infus.&blood: Hydroxyethyl starch | 1000 | ml |
|  | inhal.anesth.: Sevofluran / Sevorane | 5 | % |
|  | sedat.&analg.: Novamin / Novalgin | 2500 | mg |
|  | musc.relax.: Norcuron / Vecuronium | 2 | mg |
|  | opioid: Sufenta mite | 0.01 | mg |
|  | inhal.anesth.: Sevofluran / Sevorane | 4 | % |
|  | opioid: Sufenta mite | 0.01 | mg |
|  | infus.&blood: Packed red blood cells | 1 | n/a |
|  | musc.relax.: Norcuron / Vecuronium | 2 | mg |
|  | infus.&blood: Potassium chloride 7.45% | 20 | mval |
|  | infus.&blood: Packed red blood cells | 1 | n/a |
| NC32 | sedat.&analg.: Tranxilium | 25 | mg |
|  | sedat.&analg.: Dormicum | 7.5 | mg |
|  | infus.&blood: Ringer solution | 1500 | ml |
|  | antibiosis: Metronidazole / Clont | 500 | mg |
|  | antibiosis: Basocef / Cefazolin / Elzogram | 2000 | mg |
|  | loc.anesth.: Scandicain | 30 | mg |
|  | cardio: Atropine | 0.25 | mg |
|  | opioid: Sufenta mite | 0.03 | mg |
|  | cardio: Atropine | 0.25 | mg |
|  | sedat.&analg.: Propofol 2 % | 250 | mg |
|  | inhal.anesth.: Sevofluran / Sevorane | 2.2 | % |
|  | musc.relax.: Norcuron / Vecuronium | 10 | mg |
|  | cardio: Akrinor | 50 | mg |
|  | infus.&blood: Hydroxyethyl starch | 1000 | ml |
|  | opioid: Sufenta mite | 0.01 | mg |
|  | loc.anesth.: Naropin | 37.5 | mg |
|  | cardio: Akrinor | 50 | mg |
|  | infus.&blood: Potassium chloride 7.45% | 20 | mval |
|  | inhal.anesth.: Sevofluran / Sevorane | 1 | % |
|  | musc.relax.: Norcuron / Vecuronium | 3 | mg |
|  | opioid: Sufenta mite | 0.01 | mg |
|  | inhal.anesth.: Sevofluran / Sevorane | 1.2 | % |
|  | opioid: Sufenta mite | 0.01 | mg |
|  | infus.&blood: Packed red blood cells | 1 | n/a |
|  | musc.relax.: Norcuron / Vecuronium | 2 | mg |
| NC33 | sedat.&analg.: Tranxilium | 25 | mg |
|  | cardio: Akrinor | 50 | mg |
|  | sedat.&analg.: Dormicum | 7.5 | mg |
|  | infus.&blood: Ringer solution | 1500 | ml |
|  | infus.&blood: Potassium chloride 7.45% | 20 | mval |
|  | inhal.anesth.: Sevofluran / Sevorane | 2 | % |
|  | musc.relax.: Pantolax / Lysthenon / Succinylcholine | 100 | mg |
|  | sedat.&analg.: Propofol 2 % | 250 | mg |
|  | opioid: Sufenta mite | 0.04 | mg |
|  | antibiosis: Metronidazole / Clont | 500 | mg |
|  | antibiosis: Basocef / Cefazolin / Elzogram | 2000 | mg |
|  | cardio: Atropine | 0.5 | mg |
|  | infus.&blood: Hydroxyethyl starch | 1000 | ml |
|  | cardio: Akrinor | 50 | mg |
|  | musc.relax.: Norcuron / Vecuronium | 8 | mg |
|  | inhal.anesth.: Sevofluran / Sevorane | 1.5 | % |
|  | musc.relax.: Norcuron / Vecuronium | 2 | mg |
|  | musc.relax.: Norcuron / Vecuronium | 2 | mg |
|  | cardio: Arterenolperfusor | 0.16 | mg/h |
|  | inhal.anesth.: Sevofluran / Sevorane | 1.6 | % |
|  | sedat.&analg.: Novamin / Novalgin | 2500 | mg |
|  | cardio: Arterenolperfusor | 0.24 | mg/h |
|  | cardio: Arterenolperfusor | 0.2 | mg/h |
|  | cardio: Arterenolperfusor | 0.16 | mg/h |
| NC34 | sedat.&analg.: Tranxilium | 25 | mg |
|  | sedat.&analg.: Dormicum | 7.5 | mg |
|  | musc.relax.: Norcuron / Vecuronium | 8 | mg |
|  | infus.&blood: Ringeracetat | 1000 | ml |
|  | infus.&blood: Potassium chloride 7.45% | 20 | mval |
|  | infus.&blood: Hydroxyethyl starch | 500 | ml |
|  | antibiosis: Basocef / Cefazolin / Elzogram | 2000 | mg |
|  | loc.anesth.: Naropin | 15 | mg |
|  | sedat.&analg.: Propofol 2 % | 200 | mg |
|  | inhal.anesth.: Sevofluran / Sevorane | 0.4 | % |
|  | opioid: Sufenta mite | 0.025 | mg |
|  | antibiosis: Metronidazole / Clont | 500 | mg |
|  | inhal.anesth.: Sevofluran / Sevorane | 0.5 | % |
|  | inhal.anesth.: Sevofluran / Sevorane | 0.8 | % |
|  | loc.anesth.: Naropin | 15 | mg |
|  | loc.anesth.: Sufenta | 0.004 | mg |
|  | inhal.anesth.: Sevofluran / Sevorane | 0.5 | % |
|  | loc.anesth.: Naropin | 15 | mg |
|  | loc.anesth.: Sufenta | 0.004 | mg |
|  | opioid: Sufenta mite | 0.015 | mg |
|  | musc.relax.: Norcuron / Vecuronium | 4 | mg |
|  | loc.anesth.: Naropin | 7.5 | mg |
|  | loc.anesth.: Sufenta | 0.002 | mg |
|  | inhal.anesth.: Sevofluran / Sevorane | 1.6 | % |
|  | cardio: Akrinor | 50 | mg |
|  | inhal.anesth.: Sevofluran / Sevorane | 1.5 | % |
|  | cardio: Akrinor | 50 | mg |
|  | opioid: Sufenta mite | 0.025 | mg |
| NC35 | sedat.&analg.: Tranxilium | 25 | mg |
|  | sedat.&analg.: Dormicum | 7.5 | mg |
|  | infus.&blood: Ringer solution | 2000 | ml |
|  | musc.relax.: Norcuron / Vecuronium | 8 | mg |
|  | sedat.&analg.: Propofol 2 % | 150 | mg |
|  | opioid: Sufenta mite | 0.025 | mg |
|  | loc.anesth.: Naropin | 22.5 | mg |
|  | antibiosis: Basocef / Cefazolin / Elzogram | 2000 | mg |
|  | antibiosis: Metronidazole / Clont | 500 | mg |
|  | infus.&blood: Hydroxyethyl starch | 1000 | ml |
|  | cardio: Akrinor | 100 | mg |
|  | cardio: Atropine | 0.5 | mg |
|  | cardio: Arterenolperfusor | 0.24 | mg/h |
|  | cardio: Akrinor | 100 | mg |
|  | loc.anesth.: Naropin | 52.5 | mg |
|  | infus.&blood: Potassium chloride 7.45% | 20 | mval |
|  | cardio: Arterenolperfusor | 0.08 | mg/h |
|  | cardio: Arterenolperfusor | 0.16 | mg/h |
|  | inhal.anesth.: Sevofluran / Sevorane | 1.1 | % |
|  | cardio: Arterenolperfusor | 0.32 | mg/h |
|  | opioid: Sufenta mite | 0.015 | mg |
|  | musc.relax.: Norcuron / Vecuronium | 4 | mg |
|  | cardio: Arterenolperfusor | 0.56 | mg/h |
|  | cardio: Arterenolperfusor | 0.48 | mg/h |
|  | gastrointest.&antihist: Buscopan / Butylscopalamine | 10 | mg |
|  | gastrointest.&antihist: Buscopan / Butylscopalamine | 10 | mg |
| NC36 | sedat.&analg.: Tranxilium | 25 | mg |
|  | sedat.&analg.: Dormicum | 7.5 | mg |
|  | diuretic&corticost.: Hydrocortison | 100 | mg |
|  | infus.&blood: Ringer solution | 1000 | ml |
|  | opioid: Sufenta mite | 0.03 | mg |
|  | sedat.&analg.: Propofol 2 % | 150 | mg |
|  | musc.relax.: Norcuron / Vecuronium | 6 | mg |
|  | loc.anesth.: Scandicain | 30 | mg |
|  | inhal.anesth.: Sevofluran / Sevorane | 1.5 | % |
|  | antibiosis: Metronidazole / Clont | 500 | mg |
|  | antibiosis: Basocef / Cefazolin / Elzogram | 2000 | mg |
|  | cardio: Akrinor | 50 | mg |
|  | cardio: Akrinor | 50 | mg |
|  | cardio: Atropine | 0.25 | mg |
|  | infus.&blood: Hydroxyethyl starch | 1500 | ml |
|  | cardio: Atropine | 0.25 | mg |
|  | diuretic&corticost.: Hydrocortison | 1.95 | mg/h |
|  | sedat.&analg.: Propofol 2 % | 100 | mg |
|  | loc.anesth.: Naropin | 37.5 | mg |
|  | opioid: Sufenta mite | 0.01 | mg |
|  | musc.relax.: Norcuron / Vecuronium | 2 | mg |
|  | cardio: Akrinor | 50 | mg |
|  | cardio: Akrinor | 50 | mg |
|  | cardio: Akrinor | 50 | mg |
|  | opioid: Sufenta mite | 0.01 | mg |
|  | musc.relax.: Norcuron / Vecuronium | 2 | mg |
| NC37 | sedat.&analg.: Tranxilium | 25 | mg |
|  | sedat.&analg.: Dormicum | 7.5 | mg |
|  | infus.&blood: Ringer solution | 1000 | ml |
|  | antibiosis: Metronidazole / Clont | 500 | mg |
|  | antibiosis: Basocef / Cefazolin / Elzogram | 2000 | mg |
|  | loc.anesth.: Scandicain | 30 | mg |
|  | inhal.anesth.: Sevofluran / Sevorane | 2.4 | % |
|  | musc.relax.: Norcuron / Vecuronium | 8 | mg |
|  | sedat.&analg.: Propofol 2 % | 200 | mg |
|  | opioid: Sufenta mite | 0.04 | mg |
|  | cardio: Akrinor | 50 | mg |
|  | cardio: Akrinor | 50 | mg |
|  | cardio: Atropine | 0.25 | mg |
|  | cardio: Akrinor | 50 | mg |
|  | infus.&blood: Voluven | 1500 | ml |
|  | loc.anesth.: Naropin | 37.5 | mg |
|  | musc.relax.: Norcuron / Vecuronium | 2 | mg |
|  | opioid: Sufenta mite | 0.01 | mg |
|  | cardio: Akrinor | 50 | mg |
| NC38 | infus.&blood: Packed red blood cells | 2 | n/a |
|  | sedat.&analg.: Tranxilium | 25 | mg |
|  | gastrointest.&antihist: Zofran | 8 | mg |
|  | sedat.&analg.: Dormicum | 7.5 | mg |
|  | infus.&blood: Ringeracetat | 1500 | ml |
|  | infus.&blood: Potassium chloride 7.45% | 20 | mval |
|  | musc.relax.: Norcuron / Vecuronium | 8 | mg |
|  | antibiosis: Metronidazole / Clont | 500 | mg |
|  | antibiosis: Basocef / Cefazolin / Elzogram | 2000 | mg |
|  | infus.&blood: Hydroxyethyl starch | 1000 | ml |
|  | sedat.&analg.: Dormicum | 2 | mg |
|  | sedat.&analg.: Propofol 2 % | 200 | mg |
|  | loc.anesth.: Naropin | 22.5 | mg |
|  | sedat.&analg.: Propofolperfusor | 280 | mg/h |
|  | opioid: Sufenta mite | 0.025 | mg |
|  | sedat.&analg.: Dormicum | 3 | mg |
|  | loc.anesth.: Naropin | 15 | mg |
|  | loc.anesth.: Naropin | 11.25 | mg |
|  | loc.anesth.: Naropin | 11.25 | mg |
|  | opioid: Sufenta mite | 0.005 | mg |
|  | sedat.&analg.: Propofolperfusor | 380 | mg/h |
|  | musc.relax.: Norcuron / Vecuronium | 2 | mg |
|  | sedat.&analg.: Novamin / Novalgin | 2500 | mg |
|  | opioid: Sufenta mite | 0.025 | mg |
|  | cardio: Akrinor | 50 | mg |
|  | opioid: Sufenta mite | 0.0125 | mg |
| NC39 | sedat.&analg.: Tranxilium | 25 | mg |
|  | sedat.&analg.: Dormicum | 7.5 | mg |
|  | infus.&blood: Potassium chloride 7.45% | 20 | mval |
|  | infus.&blood: Ringeracetat | 1000 | ml |
|  | loc.anesth.: Naropin | 22.5 | mg |
|  | antibiosis: Metronidazole / Clont | 500 | mg |
|  | opioid: Sufenta mite | 0.01 | mg |
|  | sedat.&analg.: Propofol 2 % | 200 | mg |
|  | musc.relax.: Norcuron / Vecuronium | 8 | mg |
|  | antibiosis: Basocef / Cefazolin / Elzogram | 2000 | mg |
|  | inhal.anesth.: Sevofluran / Sevorane | 2.5 | % |
|  | sedat.&analg.: Dormicum | 3 | mg |
|  | loc.anesth.: Naropin | 37.5 | mg |
|  | infus.&blood: Hydroxyethyl starch | 1000 | ml |
|  | cardio: Akrinor | 100 | mg |
|  | musc.relax.: Norcuron / Vecuronium | 2 | mg |
|  | cardio: Arterenolperfusor | 0.08 | mg/h |
|  | cardio: Arterenolperfusor | 0.16 | mg/h |
|  | cardio: Arterenolperfusor | 0.24 | mg/h |
|  | cardio: Arterenolperfusor | 0.12 | mg/h |
|  | musc.relax.: Norcuron / Vecuronium | 2 | mg |
|  | gastrointest.&antihist: Buscopan / Butylscopalamine | 20 | mg |
|  | loc.anesth.: Naropin | 37.5 | mg |
| NC40 | sedat.&analg.: Tranxilium | 10 | mg |
|  | sedat.&analg.: Dormicum | 3.75 | mg |
|  | sedat.&analg.: Propofol 2 % | 150 | mg |
|  | opioid: Sufenta mite | 0.01 | mg |
|  | musc.relax.: Norcuron / Vecuronium | 4 | mg |
|  | antibiosis: Metronidazole / Clont | 500 | mg |
|  | inhal.anesth.: Sevofluran / Sevorane | 0.6 | % |
|  | infus.&blood: Hydroxyethyl starch | 1000 | ml |
|  | sedat.&analg.: Dormicum | 3 | mg |
|  | infus.&blood: Ringeracetat | 500 | ml |
|  | antibiosis: Basocef / Cefazolin / Elzogram | 2000 | mg |
|  | infus.&blood: Potassium chloride 7.45% | 20 | mval |
|  | inhal.anesth.: Sevofluran / Sevorane | 4 | % |
|  | infus.&blood: Nabi / Sodium bicarbonate | 100 | ml |
|  | musc.relax.: Norcuron / Vecuronium | 2 | mg |
|  | cardio: Akrinor | 100 | mg |
|  | infus.&blood: Glucose 5% | 500 | ml |
|  | opioid: Sufenta mite | 0.01 | mg |
|  | cardio: Akrinor | 50 | mg |
|  | cardio: Akrinor | 50 | mg |
|  | infus.&blood: Nabi / Sodium bicarbonate | 2500 | ml |
|  | musc.relax.: Norcuron / Vecuronium | 2 | mg |
|  | gastrointest.&antihist: Buscopan / Butylscopalamine | 20 | mg |
| NC41 | infus.&blood: Packed red blood cells | 3 | n/a |
|  | sedat.&analg.: Tranxilium | 10 | mg |
|  | gastrointest.&antihist: Zofran | 8 | mg |
|  | sedat.&analg.: Dormicum | 7.5 | mg |
|  | loc.anesth.: Naropin | 22.5 | mg |
|  | antibiosis: Metronidazole / Clont | 500 | mg |
|  | sedat.&analg.: Propofol 2 % | 200 | mg |
|  | opioid: Sufenta mite | 0.01 | mg |
|  | musc.relax.: Norcuron / Vecuronium | 6 | mg |
|  | sedat.&analg.: Dormicum | 3 | mg |
|  | infus.&blood: Ringeracetat | 1000 | ml |
|  | infus.&blood: Potassium chloride 7.45% | 20 | mval |
|  | antibiosis: Basocef / Cefazolin / Elzogram | 2000 | mg |
|  | inhal.anesth.: Sevofluran / Sevorane | 2 | % |
|  | loc.anesth.: Naropin | 37.5 | mg |
|  | musc.relax.: Norcuron / Vecuronium | 2 | mg |
|  | infus.&blood: Hydroxyethyl starch | 1000 | ml |
|  | cardio: Arterenolperfusor | 0.08 | mg/h |
|  | musc.relax.: Norcuron / Vecuronium | 2 | mg |
|  | infus.&blood: Packed red blood cells | 1 | n/a |
|  | cardio: Akrinor | 50 | mg |
|  | cardio: Akrinor | 50 | mg |
|  | musc.relax.: Norcuron / Vecuronium | 2 | mg |
| NC42 | sedat.&analg.: Tranxilium | 25 | mg |
|  | sedat.&analg.: Dormicum | 7.5 | mg |
|  | opioid: Sufenta mite | 0.01 | mg |
|  | sedat.&analg.: Propofol 2 % | 200 | mg |
|  | antibiosis: Metronidazole / Clont | 500 | mg |
|  | musc.relax.: Norcuron / Vecuronium | 6 | mg |
|  | inhal.anesth.: Sevofluran / Sevorane | 0.6 | % |
|  | antibiosis: Basocef / Cefazolin / Elzogram | 2000 | mg |
|  | infus.&blood: Ringeracetat | 1000 | ml |
|  | cardio: Akrinor | 50 | mg |
|  | infus.&blood: Potassium chloride 7.45% | 20 | mval |
|  | infus.&blood: Hydroxyethyl starch | 1000 | ml |
|  | sedat.&analg.: Dormicum | 3 | mg |
|  | musc.relax.: Norcuron / Vecuronium | 2 | mg |
|  | inhal.anesth.: Sevofluran / Sevorane | 5 | % |
|  | cardio: Akrinor | 50 | mg |
|  | inhal.anesth.: Sevofluran / Sevorane | 4 | % |
|  | musc.relax.: Norcuron / Vecuronium | 2 | mg |
|  | opioid: Sufenta mite | 0.015 | mg |
|  | inhal.anesth.: Sevofluran / Sevorane | 1.3 | % |
|  | sedat.&analg.: Novamin / Novalgin | 2500 | mg |
|  | inhal.anesth.: Sevofluran / Sevorane | 5 | % |
|  | musc.relax.: Norcuron / Vecuronium | 2 | mg |
|  | inhal.anesth.: Sevofluran / Sevorane | 1.8 | % |
|  | opioid: Sufenta mite | 0.01 | mg |
|  | gastrointest.&antihist: Buscopan / Butylscopalamine | 10 | mg |
| NC43 | sedat.&analg.: Tranxilium | 25 | mg |
|  | sedat.&analg.: Dormicum | 7.5 | mg |
|  | antibiosis: Metronidazole / Clont | 500 | mg |
|  | loc.anesth.: Naropin | 22.5 | mg |
|  | sedat.&analg.: Propofol 2 % | 200 | mg |
|  | musc.relax.: Norcuron / Vecuronium | 6 | mg |
|  | opioid: Sufenta mite | 0.01 | mg |
|  | antibiosis: Basocef / Cefazolin / Elzogram | 2000 | mg |
|  | inhal.anesth.: Sevofluran / Sevorane | 0.6 | % |
|  | sedat.&analg.: Dormicum | 3 | mg |
|  | infus.&blood: Ringeracetat | 1000 | ml |
|  | infus.&blood: Potassium chloride 7.45% | 20 | mval |
|  | inhal.anesth.: Sevofluran / Sevorane | 2.5 | % |
|  | loc.anesth.: Naropin | 37.5 | mg |
|  | cardio: Akrinor | 50 | mg |
|  | inhal.anesth.: Sevofluran / Sevorane | 0.6 | % |
|  | infus.&blood: Hydroxyethyl starch | 1000 | ml |
|  | inhal.anesth.: Sevofluran / Sevorane | 2 | % |
|  | opioid: Sufenta mite | 0.015 | mg |
|  | musc.relax.: Norcuron / Vecuronium | 2 | mg |
|  | cardio: Akrinor | 50 | mg |
|  | musc.relax.: Norcuron / Vecuronium | 2 | mg |
|  | cardio: Akrinor | 50 | mg |
|  | cardio: Akrinor | 50 | mg |
|  | cardio: Arterenolperfusor | 0.08 | mg/h |
| NC46 | sedat.&analg.: Tranxilium | 25 | mg |
|  | sedat.&analg.: Dormicum | 7.5 | mg |
|  | infus.&blood: Ringeracetat | 1500 | ml |
|  | infus.&blood: Potassium chloride 7.45% | 20 | mval |
|  | infus.&blood: Hydroxyethyl starch | 1000 | ml |
|  | sedat.&analg.: Propofol 2 % | 100 | mg |
|  | loc.anesth.: Naropin | 22.5 | mg |
|  | opioid: Sufenta mite | 0.015 | mg |
|  | sedat.&analg.: Propofolperfusor | 300 | mg/h |
|  | musc.relax.: Norcuron / Vecuronium | 8 | mg |
|  | sedat.&analg.: Propofolperfusor | 400 | mg/h |
|  | loc.anesth.: Sufenta | 0.01 | mg |
|  | loc.anesth.: Naropin | 15 | mg |
|  | loc.anesth.: Naropin | 11.25 | mg |
|  | opioid: Sufenta mite | 0.01 | mg |
|  | musc.relax.: Norcuron / Vecuronium | 8 | mg |
|  | opioid: Sufenta mite | 0.015 | mg |
|  | musc.relax.: Norcuron / Vecuronium | 4 | mg |
| NC47 | sedat.&analg.: Tranxilium | 10 | mg |
|  | sedat.&analg.: Dormicum | 3.75 | mg |
|  | infus.&blood: Ringeracetat | 1000 | ml |
|  | antibiosis: Metronidazole / Clont | 500 | mg |
|  | antibiosis: Basocef / Cefazolin / Elzogram | 2000 | mg |
|  | sedat.&analg.: Propofol 2 % | 160 | mg |
|  | opioid: Sufenta mite | 0.02 | mg |
|  | musc.relax.: Norcuron / Vecuronium | 6 | mg |
|  | inhal.anesth.: Sevofluran / Sevorane | 0.5 | % |
|  | cardio: Akrinor | 50 | mg |
|  | inhal.anesth.: Sevofluran / Sevorane | 1.1 | % |
|  | cardio: Atropine | 0.5 | mg |
|  | infus.&blood: Hydroxyethyl starch | 500 | ml |
|  | cardio: Akrinor | 50 | mg |
|  | inhal.anesth.: Sevofluran / Sevorane | 0.9 | % |
|  | loc.anesth.: Naropin | 75 | mg |
|  | inhal.anesth.: Sevofluran / Sevorane | 1.1 | % |
| NC48 | sedat.&analg.: Tranxilium | 25 | mg |
|  | sedat.&analg.: Dormicum | 7.5 | mg |
|  | infus.&blood: Ringer solution | 2000 | ml |
|  | antibiosis: Metronidazole / Clont | 500 | mg |
|  | antibiosis: Basocef / Cefazolin / Elzogram | 2000 | mg |
|  | loc.anesth.: Scandicain | 30 | mg |
|  | cardio: Akrinor | 50 | mg |
|  | inhal.anesth.: Sevofluran / Sevorane | 1.5 | % |
|  | musc.relax.: Pantolax / Lysthenon / Succinylcholine | 100 | mg |
|  | opioid: Sufenta mite | 0.04 | mg |
|  | sedat.&analg.: Propofol 2 % | 200 | mg |
|  | cardio: Akrinor | 50 | mg |
|  | musc.relax.: Norcuron / Vecuronium | 6 | mg |
|  | loc.anesth.: Naropin | 37.5 | mg |
|  | infus.&blood: Hydroxyethyl starch | 500 | ml |
|  | cardio: Atropine | 0.25 | mg |
|  | musc.relax.: Norcuron / Vecuronium | 2 | mg |
|  | musc.relax.: Norcuron / Vecuronium | 6 | mg |
|  | cardio: Akrinor | 50 | mg |
|  | cardio: Arterenolperfusor | 0.08 | mg/h |
| NL1 | n/a | n/a | n/a |
| NL2 | n/a | n/a | n/a |
| NL3 | sedat.&analg.: Dormicum | 3.75 | mg |
|  | infus.&blood: Sterofundin 1/1 E | 3500 | ml |
|  | inhal.anesth.: Sevofluran / Sevorane | 1.5 | % |
|  | musc.relax.: Esmeron / Rocuronium | 50 | mg |
|  | antibiosis: Cefuroxime | 1500 | mg |
|  | opioid: Sufenta mite | 0.04 | mg |
|  | sedat.&analg.: Propofol | 150 | mg |
|  | cardio: Arterenol | 0.01 | mg |
|  | cardio: Arterenol | 0.02 | mg |
|  | cardio: Arterenol | 0.02 | mg |
|  | cardio: Arterenol | 0.005 | mg |
|  | cardio: Atropine | 1 | mg |
|  | cardio: Atropine | 0.5 | mg |
|  | cardio: Arterenolperfusor | 0.3 | mg/h |
|  | antibiosis: Metronidazole / Clont | 500 | mg |
|  | cardio: Arterenolperfusor | 0.48 | mg/h |
|  | cardio: Arterenolperfusor | 0.42 | mg/h |
|  | opioid: Sufenta mite | 0.02 | mg |
|  | inhal.anesth.: Sevofluran / Sevorane | 1.7 | % |
|  | cardio: Arterenolperfusor | 0.3 | mg/h |
|  | cardio: Arterenolperfusor | 0.36 | mg/h |
|  | opioid: Sufenta mite | 0.01 | mg |
|  | cardio: Arterenolperfusor | 0.24 | mg/h |
|  | inhal.anesth.: Sevofluran / Sevorane | 2 | % |
|  | cardio: Arterenolperfusor | 0.36 | mg/h |
|  | musc.relax.: Esmeron / Rocuronium | 10 | mg |
|  | opioid: Sufenta mite | 0.01 | mg |
|  | cardio: Arterenolperfusor | 0.42 | mg/h |
|  | cardio: Arterenolperfusor | 0.48 | mg/h |
|  | inhal.anesth.: Sevofluran / Sevorane | 1.7 | % |
|  | cardio: Arterenolperfusor | 0.36 | mg/h |
|  | inhal.anesth.: Sevofluran / Sevorane | 2.1 | % |
|  | cardio: Arterenolperfusor | 0.24 | mg/h |
|  | cardio: Arterenolperfusor | 0.3 | mg/h |
|  | inhal.anesth.: Sevofluran / Sevorane | 1.9 | % |
|  | opioid: Sufenta mite | 0.01 | mg |
|  | cardio: Arterenolperfusor | 0.36 | mg/h |
|  | inhal.anesth.: Sevofluran / Sevorane | 1.8 | % |
|  | cardio: Arterenolperfusor | 0.42 | mg/h |
|  | cardio: Arterenolperfusor | 0.48 | mg/h |
|  | opioid: Sufenta mite | 0.001 | mg |
|  | cardio: Arterenolperfusor | 0.54 | mg/h |
|  | infus.&blood: Voluven | 500 | ml |
|  | cardio: Arterenolperfusor | 0.6 | mg/h |
|  | cardio: Arterenolperfusor | 0.72 | mg/h |
| NL4 | sedat.&analg.: Dormicum | 7.5 | mg |
|  | infus.&blood: Sterofundin 1/1 E | 2500 | ml |
|  | sedat.&analg.: Propofol | 200 | mg |
|  | opioid: Sufenta mite | 0.04 | mg |
|  | musc.relax.: Esmeron / Rocuronium | 40 | mg |
|  | inhal.anesth.: Sevofluran / Sevorane | 1.5 | % |
|  | sedat.&analg.: Propofol | 50 | mg |
|  | antibiosis: Cefuroxime | 1500 | mg |
|  | cardio: Arterenolperfusor | 0.18 | mg/h |
|  | cardio: Arterenolperfusor | 0.3 | mg/h |
|  | opioid: Sufenta mite | 0.01 | mg |
|  | musc.relax.: Esmeron / Rocuronium | 10 | mg |
|  | inhal.anesth.: Sevofluran / Sevorane | 1.9 | % |
|  | cardio: Arterenolperfusor | 0.12 | mg/h |
|  | opioid: Sufenta mite | 0.02 | mg |
|  | cardio: Arterenolperfusor | 0.24 | mg/h |
|  | opioid: Sufenta mite | 0.02 | mg |
|  | cardio: Arterenolperfusor | 0.36 | mg/h |
|  | inhal.anesth.: Sevofluran / Sevorane | 2.5 | % |
|  | cardio: Arterenolperfusor | 0.42 | mg/h |
|  | cardio: Arterenolperfusor | 0.6 | mg/h |
|  | cardio: Arterenolperfusor | 0.9 | mg/h |
| NL5 | sedat.&analg.: Dormicum | 3.75 | mg |
|  | infus.&blood: Sterofundin 1/1 E | 2000 | ml |
|  | antibiosis: Cefuroxime | 1500 | mg |
|  | musc.relax.: Esmeron / Rocuronium | 50 | mg |
|  | inhal.anesth.: Sevofluran / Sevorane | 1.3 | % |
|  | opioid: Sufenta mite | 0.05 | mg |
|  | sedat.&analg.: Propofol | 200 | mg |
|  | cardio: Arterenolperfusor | 0.006 | mg/h |
|  | inhal.anesth.: Sevofluran / Sevorane | 2 | % |
|  | opioid: Sufenta mite | 0.02 | mg |
|  | opioid: Sufenta mite | 0.015 | mg |
|  | cardio: Clonidine / Catapresan | 0.15 | mg |
|  | inhal.anesth.: Sevofluran / Sevorane | 2.3 | % |
|  | cardio: Arterenolperfusor | 0.12 | mg/h |
|  | opioid: Sufenta mite | 0.03 | mg |
|  | cardio: Arterenolperfusor | 0.18 | mg/h |
|  | cardio: Arterenolperfusor | 0.12 | mg/h |
|  | opioid: Sufenta mite | 0.01 | mg |
|  | opioid: Sufenta mite | 0.01 | mg |
|  | opioid: Sufenta mite | 0.01 | mg |
| NL7 | sedat.&analg.: Dormicum | 7.5 | mg |
|  | infus.&blood: Ringer solution | 1500 | ml |
|  | opioid: Sufenta mite | 0.03 | mg |
|  | musc.relax.: Esmeron / Rocuronium | 30 | mg |
|  | sedat.&analg.: Propofol 2 % | 200 | mg |
|  | antibiosis: Metronidazole / Clont | 500 | mg |
|  | antibiosis: Cefuroxime | 1500 | mg |
|  | inhal.anesth.: Sevofluran / Sevorane | 2.4 | % |
|  | musc.relax.: Esmeron / Rocuronium | 20 | mg |
|  | opioid: Sufenta mite | 0.02 | mg |
|  | opioid: Sufenta mite | 0.01 | mg |
|  | cardio: Arterenolperfusor | 0.3 | mg/h |
|  | musc.relax.: Esmeron / Rocuronium | 10 | mg |
|  | cardio: Arterenolperfusor | 0.24 | mg/h |
|  | inhal.anesth.: Sevofluran / Sevorane | 2.2 | % |
|  | cardio: Arterenolperfusor | 0.18 | mg/h |
|  | cardio: Arterenolperfusor | 0.12 | mg/h |
|  | infus.&blood: Voluven | 500 | ml |
|  | cardio: Arterenolperfusor | 0.06 | mg/h |
|  | inhal.anesth.: Sevofluran / Sevorane | 1.8 | % |
|  | cardio: Arterenolperfusor | 0.006 | mg/h |
|  | opioid: Sufenta mite | 0.025 | mg |
|  | cardio: Arterenolperfusor | 0.06 | mg/h |
|  | opioid: Sufenta mite | 0.015 | mg |
|  | inhal.anesth.: Sevofluran / Sevorane | 2 | % |
|  | musc.relax.: Esmeron / Rocuronium | 10 | mg |
|  | cardio: Arterenolperfusor | 0.12 | mg/h |
|  | cardio: Arterenolperfusor | 0.006 | mg/h |
| NL8 | sedat.&analg.: Dormicum | 3.75 | mg |
|  | antibiosis: Cefuroxime | 1500 | mg |
|  | infus.&blood: Ringer solution | 1500 | ml |
|  | opioid: Sufenta mite | 0.04 | mg |
|  | sedat.&analg.: Propofol | 130 | mg |
|  | inhal.anesth.: Sevofluran / Sevorane | 1.5 | % |
|  | cardio: Arterenolperfusor | 0.18 | mg/h |
|  | musc.relax.: Esmeron / Rocuronium | 35 | mg |
|  | cardio: Arterenol | 0.01 | mg |
|  | cardio: Arterenolperfusor | 0.06 | mg/h |
|  | inhal.anesth.: Sevofluran / Sevorane | 1.8 | % |
|  | cardio: Arterenolperfusor | 0.006 | mg/h |
|  | musc.relax.: Esmeron / Rocuronium | 15 | mg |
|  | opioid: Sufenta mite | 0.02 | mg |
|  | infus.&blood: Hydroxyethyl starch | 500 | ml |
|  | opioid: Sufenta mite | 0.01 | mg |
|  | opioid: Sufenta mite | 0.03 | mg |
|  | cardio: Clonidine / Catapresan | 0.03 | mg |
|  | opioid: Sufenta mite | 0.02 | mg |
|  | opioid: Sufenta mite | 0.01 | mg |
|  | sedat.&analg.: Novamin / Novalgin | 1000 | mg |
|  | opioid: Sufenta mite | 0.01 | mg |
| NL9 | sedat.&analg.: Dormicum | 7.5 | mg |
|  | sedat.&analg.: Propofol | 200 | mg |
|  | opioid: Sufenta mite | 0.03 | mg |
|  | cardio: Arterenolperfusor | 1500 | mg/h |
|  | antibiosis: Cefuroxime | 1500 | mg |
|  | musc.relax.: Esmeron / Rocuronium | 30 | mg |
|  | cardio: Arterenolperfusor | 0.18 | mg/h |
|  | inhal.anesth.: Sevofluran / Sevorane | 1.5 | % |
|  | cardio: Arterenolperfusor | 0.3 | mg/h |
|  | musc.relax.: Esmeron / Rocuronium | 10 | mg |
|  | opioid: Sufenta mite | 0.02 | mg |
|  | cardio: Arterenolperfusor | 0.24 | mg/h |
|  | inhal.anesth.: Sevofluran / Sevorane | 2 | % |
|  | cardio: Arterenolperfusor | 0.18 | mg/h |
|  | opioid: Sufenta mite | 0.02 | mg |
|  | cardio: Arterenolperfusor | 0.006 | mg/h |
|  | inhal.anesth.: Sevofluran / Sevorane | 1.8 | % |
|  | opioid: Sufenta mite | 0.02 | mg |
| NL10 | n/a | n/a | n/a |
| NL12 | cardio: Arterenolperfusor | 0.06 | mg/h |
|  | cardio: Arterenolperfusor | 0.12 | mg/h |
|  | cardio: Arterenolperfusor | 0.06 | mg/h |
|  | cardio: Arterenolperfusor | 0.03 | mg/h |
|  | cardio: Arterenolperfusor | 0.06 | mg/h |
|  | infus.&blood: Ringer solution | 1000 | ml |
|  | cardio: Arterenolperfusor | 0.12 | mg/h |
|  | cardio: Arterenolperfusor | 0.18 | mg/h |
|  | cardio: Arterenolperfusor | 0.3 | mg/h |
|  | diuretic&corticost.: Furosemide / Lasix | 5 | mg |
|  | cardio: Clonidine / Catapresan | 0.75 | mg |
|  | cardio: Clonidine / Catapresan | 0.75 | mg |
|  | antibiosis: Cefuroxime | 1500 | mg |
|  | inhal.anesth.: Sevofluran / Sevorane | 1.5 | % |
|  | inhal.anesth.: Sevofluran / Sevorane | 1.7 | % |
|  | musc.relax.: Esmeron / Rocuronium | 10 | mg |
|  | musc.relax.: Esmeron / Rocuronium | 10 | mg |
|  | musc.relax.: Esmeron / Rocuronium | 10 | mg |
|  | musc.relax.: Esmeron / Rocuronium | 50 | mg |
|  | opioid: Sufenta mite | 0.01 | mg |
|  | opioid: Sufenta mite | 0.015 | mg |
|  | sedat.&analg.: Propofol | 150 | mg |
|  | sedat.&analg.: Dormicum | 7.5 | mg |
|  | opioid: Sufenta mite | 0.01 | mg |
|  | sedat.&analg.: Novamin / Novalgin | 1000 | mg |
|  | opioid: Sufenta mite | 0.04 | mg |
| NL14 | sedat.&analg.: Dalmadorm | 30 | mg |
|  | sedat.&analg.: Dormicum | 7.5 | mg |
|  | antibiosis: Cefuroxime | 1500 | mg |
|  | infus.&blood: Ringer solution | 2000 | ml |
|  | sedat.&analg.: Propofol | 150 | mg |
|  | opioid: Sufenta mite | 0.04 | mg |
|  | musc.relax.: Esmeron / Rocuronium | 40 | mg |
|  | inhal.anesth.: Sevofluran / Sevorane | 1.4 | % |
|  | diuretic&corticost.: Dexamethasone / Fortecortin | 4 | mg |
|  | gastrointest.&antihist: Zofran | 4 | mg |
|  | cardio: Arterenolperfusor | 0.6 | mg/h |
|  | cardio: Arterenolperfusor | 0.3 | mg/h |
|  | opioid: Sufenta mite | 0.01 | mg |
|  | cardio: Arterenolperfusor | 0.24 | mg/h |
|  | musc.relax.: Esmeron / Rocuronium | 10 | mg |
|  | opioid: Sufenta mite | 0.01 | mg |
|  | cardio: Arterenolperfusor | 0.18 | mg/h |
|  | inhal.anesth.: Sevofluran / Sevorane | 1.5 | % |
|  | opioid: Sufenta mite | 0.015 | mg |
|  | inhal.anesth.: Sevofluran / Sevorane | 1.6 | % |
|  | cardio: Clonidine / Catapresan | 0.4 | mg |
|  | cardio: Clonidine / Catapresan | 0.3 | mg |
|  | inhal.anesth.: Sevofluran / Sevorane | 1.5 | % |
|  | musc.relax.: Esmeron / Rocuronium | 5 | mg |
|  | opioid: Sufenta mite | 0.01 | mg |
|  | inhal.anesth.: Sevofluran / Sevorane | 1.8 | % |
|  | cardio: Clonidine / Catapresan | 0.6 | mg |
|  | cardio: Arterenolperfusor | 0.12 | mg/h |
|  | infus.&blood: Packed red blood cells | 1 | n/a |
|  | inhal.anesth.: Sevofluran / Sevorane | 1.5 | % |
|  | infus.&blood: Voluven | 1000 | ml |
|  | inhal.anesth.: Sevofluran / Sevorane | 1.6 | % |
|  | cardio: Arterenolperfusor | 0.09 | mg/h |
|  | cardio: Clonidine / Catapresan | 0.15 | mg |
|  | inhal.anesth.: Sevofluran / Sevorane | 1.5 | % |
|  | opioid: Sufenta mite | 0.005 | mg |
| NL17 | sedat.&analg.: Dormicum | 7.5 | mg |
|  | infus.&blood: Sterofundin 1/1 E | 5000 | ml |
|  | opioid: Sufenta mite | 0.04 | mg |
|  | sedat.&analg.: Propofol 1 % | 150 | mg |
|  | antibiosis: Metronidazole / Clont | 500 | mg |
|  | antibiosis: Cefuroxime | 1500 | mg |
|  | musc.relax.: Esmeron / Rocuronium | 50 | mg |
|  | inhal.anesth.: Sevofluran / Sevorane | 1.4 | % |
|  | cardio: Arterenolperfusor | 0.9 | mg/h |
|  | cardio: Arterenolperfusor | 0.6 | mg/h |
|  | inhal.anesth.: Sevofluran / Sevorane | 1.9 | % |
|  | cardio: Arterenolperfusor | 0.48 | mg/h |
|  | cardio: Arterenolperfusor | 0.6 | mg/h |
|  | opioid: Sufenta mite | 0.02 | mg |
|  | infus.&blood: Voluven | 2500 | ml |
|  | cardio: Arterenolperfusor | 0.66 | mg/h |
|  | opioid: Sufenta mite | 0.01 | mg |
|  | opioid: Sufenta mite | 0.01 | mg |
|  | cardio: Arterenolperfusor | 0.48 | mg/h |
|  | musc.relax.: Esmeron / Rocuronium | 10 | mg |
|  | cardio: Arterenolperfusor | 0.6 | mg/h |
|  | opioid: Sufenta mite | 0.01 | mg |
|  | cardio: Arterenolperfusor | 0.48 | mg/h |
|  | cardio: Arterenolperfusor | 0.36 | mg/h |
|  | cardio: Arterenolperfusor | 0.48 | mg/h |
|  | opioid: Sufenta mite | 0.01 | mg |
|  | cardio: Arterenolperfusor | 0.54 | mg/h |
|  | cardio: Arterenolperfusor | 0.42 | mg/h |
|  | antibiosis: Cefuroxime | 1500 | mg |
|  | antibiosis: Metronidazole / Clont | 500 | mg |
|  | musc.relax.: Esmeron / Rocuronium | 10 | mg |
|  | cardio: Arterenolperfusor | 0.3 | mg/h |
|  | cardio: Arterenolperfusor | 0.42 | mg/h |
|  | opioid: Sufenta mite | 0.01 | mg |
|  | cardio: Arterenolperfusor | 0.3 | mg/h |
|  | cardio: Arterenolperfusor | 0.48 | mg/h |
|  | cardio: Arterenolperfusor | 0.24 | mg/h |
|  | opioid: Sufenta mite | 0.015 | mg |
|  | cardio: Arterenolperfusor | 0.6 | mg/h |
|  | cardio: Arterenolperfusor | 0.36 | mg/h |
|  | musc.relax.: Esmeron / Rocuronium | 10 | mg |
|  | cardio: Arterenolperfusor | 0.42 | mg/h |
|  | cardio: Arterenolperfusor | 0.48 | mg/h |
|  | musc.relax.: Esmeron / Rocuronium | 10 | mg |
|  | cardio: Arterenolperfusor | 0.6 | mg/h |
|  | opioid: Sufenta mite | 0.01 | mg |
|  | cardio: Arterenolperfusor | 0.72 | mg/h |
|  | infus.&blood: Packed red blood cells | 1 | n/a |
|  | musc.relax.: Esmeron / Rocuronium | 20 | mg |
| NL18 | gastrointest.&antihist.: Zantic | 300 | mg |
|  | gastrointest.&antihist.: Zantic | 150 | mg |
|  | sedat.&analg.: Dormicum | 3.75 | mg |
|  | infus.&blood: Sterofundin 1/1 E | 1500 | ml |
|  | opioid: Sufenta mite | 0.04 | mg |
|  | musc.relax.: Esmeron / Rocuronium | 50 | mg |
|  | sedat.&analg.: Propofol 1 % | 180 | mg |
|  | inhal.anesth.: Sevofluran / Sevorane | 1.6 | % |
|  | antibiosis: Metronidazole / Clont | 500 | mg |
|  | antibiosis: Cefuroxime | 1500 | mg |
|  | infus.&blood: Voluven | 1000 | ml |
|  | cardio: Arterenolperfusor | 0.6 | mg/h |
|  | opioid: Sufenta mite | 0.02 | mg |
|  | cardio: Arterenolperfusor | 0.3 | mg/h |
|  | opioid: Sufenta mite | 0.01 | mg |
|  | cardio: Arterenolperfusor | 0.006 | mg/h |
|  | cardio: Arterenolperfusor | 0.12 | mg/h |
|  | cardio: Arterenolperfusor | 0.006 | mg/h |
|  | opioid: Sufenta mite | 0.01 | mg |
|  | cardio: Arterenolperfusor | 0.03 | mg/h |
|  | cardio: Arterenolperfusor | 0.06 | mg/h |
|  | cardio: Arterenolperfusor | 0.3 | mg/h |
|  | cardio: Arterenolperfusor | 0.18 | mg/h |
|  | cardio: Arterenolperfusor | 0.006 | mg/h |
|  | opioid: Sufenta mite | 0.01 | mg |
| NL21 | infus.&blood: Hydroxyethyl starch | 500 | ml |
|  | infus.&blood: Potassium chloride 7.45% | 20 | mval |
|  | infus.&blood: Ringer solution | 1000 | ml |
|  | antibiosis: Metronidazole / Clont | 500 | mg |
|  | sedat.&analg.: Propofol 2 % | 250 | mg |
|  | musc.relax.: Norcuron / Vecuronium | 8 | mg |
|  | opioid: Sufenta mite | 0.04 | mg |
|  | antibiosis: Basocef / Cefazolin / Elzogram | 2000 | mg |
|  | inhal.anesth.: Sevofluran / Sevorane | 0.5 | % |
|  | inhal.anesth.: Sevofluran / Sevorane | 1.5 | % |
|  | opioid: Sufenta mite | 0.02 | mg |
|  | musc.relax.: Norcuron / Vecuronium | 2 | mg |
|  | cardio: Arterenolperfusor | 0.4 | mg/h |
|  | inhal.anesth.: Sevofluran / Sevorane | 1.2 | % |
|  | cardio: Arterenolperfusor | 0.32 | mg/h |
|  | opioid: Sufenta mite | 0.02 | mg |
|  | cardio: Arterenolperfusor | 0.2 | mg/h |
|  | cardio: Arterenolperfusor | 0.12 | mg/h |
|  | cardio: Arterenolperfusor | 0.2 | mg/h |
|  | cardio: Arterenolperfusor | 0.16 | mg/h |
|  | cardio: Arterenolperfusor | 0.08 | mg/h |
|  | musc.relax.: Norcuron / Vecuronium | 2 | mg |
|  | cardio: Arterenolperfusor | 0.16 | mg/h |
|  | opioid: Sufenta mite | 0.02 | mg |
|  | cardio: Arterenolperfusor | 0.04 | mg/h |
|  | cardio: Arterenolperfusor | 0.12 | mg/h |
|  | cardio: Arterenolperfusor | 0.16 | mg/h |
|  | musc.relax.: Norcuron / Vecuronium | 2 | mg |
|  | opioid: Sufenta mite | 0.01 | mg |
|  | cardio: Arterenolperfusor | 0.32 | mg/h |
| NL22 | sedat.&analg.: Tranxilium | 25 | mg |
|  | sedat.&analg.: Dormicum | 7.5 | mg |
|  | antibiosis: Metronidazole / Clont | 500 | mg |
|  | loc.anesth.: Naropin | 11.25 | mg |
|  | inhal.anesth.: Sevofluran / Sevorane | 0.6 | % |
|  | musc.relax.: Norcuron / Vecuronium | 6 | mg |
|  | opioid: Sufenta mite | 0.01 | mg |
|  | sedat.&analg.: Propofol 2 % | 200 | mg |
|  | antibiosis: Basocef / Cefazolin / Elzogram | 2000 | mg |
|  | infus.&blood: Ringeracetat | 1500 | ml |
|  | sedat.&analg.: Dormicum | 3 | mg |
|  | cardio: Akrinor | 50 | mg |
|  | loc.anesth.: Naropin | 30 | mg |
|  | loc.anesth.: Sufenta | 0.01 | mg |
|  | infus.&blood: Potassium chloride 7.45% | 20 | mval |
|  | inhal.anesth.: Sevofluran / Sevorane | 3 | % |
|  | musc.relax.: Norcuron / Vecuronium | 2 | mg |
|  | cardio: Arterenolperfusor | 0.2 | mg/h |
|  | cardio: Arterenolperfusor | 0.12 | mg/h |
|  | musc.relax.: Norcuron / Vecuronium | 2 | mg |
|  | opioid: Sufenta mite | 0.01 | mg |
|  | musc.relax.: Norcuron / Vecuronium | 2 | mg |
|  | loc.anesth.: Sufenta | 0.01 | mg |
|  | sedat.&analg.: Dormicum | 2 | mg |
|  | loc.anesth.: Naropin | 30 | mg |
| NL24 | sedat.&analg.: Dormicum | 3.75 | mg |
|  | infus.&blood: Sterofundin 1/1 E | 1500 | ml |
|  | musc.relax.: Esmeron / Rocuronium | 40 | mg |
|  | opioid: Sufenta mite | 0.04 | mg |
|  | sedat.&analg.: Propofol 1 % | 200 | mg |
|  | inhal.anesth.: Sevofluran / Sevorane | 1.6 | % |
|  | cardio: Arterenolperfusor | 0.3 | mg/h |
|  | antibiosis: Cefuroxime | 1500 | mg |
|  | inhal.anesth.: Sevofluran / Sevorane | 1.9 | % |
|  | opioid: Sufenta mite | 0.01 | mg |
|  | antibiosis: Metronidazole / Clont | 500 | mg |
|  | cardio: Arterenolperfusor | 0.18 | mg/h |
|  | musc.relax.: Esmeron / Rocuronium | 10 | mg |
|  | cardio: Arterenolperfusor | 0.06 | mg/h |
|  | opioid: Sufenta mite | 0.015 | mg |
|  | inhal.anesth.: Sevofluran / Sevorane | 1.7 | % |
|  | cardio: Arterenolperfusor | 0.06 | mg/h |
|  | opioid: Sufenta mite | 0.01 | mg |
|  | musc.relax.: Esmeron / Rocuronium | 10 | mg |
|  | opioid: Sufenta mite | 0.01 | mg |
|  | cardio: Arterenolperfusor | 0.09 | mg/h |
|  | inhal.anesth.: Sevofluran / Sevorane | 1.8 | % |
|  | musc.relax.: Esmeron / Rocuronium | 10 | mg |
|  | opioid: Sufenta mite | 0.01 | mg |
|  | cardio: Arterenolperfusor | 0.015 | mg/h |
|  | cardio: Arterenolperfusor | 0.24 | mg/h |
|  | infus.&blood: Ringer solution | 500 | ml |
|  | inhal.anesth.: Sevofluran / Sevorane | 1.7 | % |
|  | inhal.anesth.: Sevofluran / Sevorane | 1.6 | % |
|  | cardio: Arterenolperfusor | 0.18 | mg/h |
|  | cardio: Arterenolperfusor | 0.24 | mg/h |
| NL25 | sedat.&analg.: Dalmadorm | 30 | mg |
|  | sedat.&analg.: Dormicum | 7.5 | mg |
|  | infus.&blood: Sterofundin 1/1 E | 3500 | ml |
|  | sedat.&analg.: Propofol 1 % | 200 | mg |
|  | antibiosis: Cefuroxime | 1500 | mg |
|  | cardio: Arterenol | 0.01 | mg |
|  | musc.relax.: Esmeron / Rocuronium | 50 | mg |
|  | opioid: Sufenta mite | 0.04 | mg |
|  | inhal.anesth.: Sevofluran / Sevorane | 1.2 | % |
|  | cardio: Arterenol | 0.01 | mg |
|  | cardio: Arterenol | 0.015 | mg |
|  | sedat.&analg.: Propofol 1 % | 50 | mg |
|  | infus.&blood: Voluven | 500 | ml |
|  | cardio: Arterenol | 0.01 | mg |
|  | cardio: Arterenolperfusor | 0.6 | mg/h |
|  | cardio: Arterenolperfusor | 0.48 | mg/h |
|  | inhal.anesth.: Sevofluran / Sevorane | 1.8 | % |
|  | cardio: Arterenolperfusor | 0.36 | mg/h |
|  | cardio: Arterenolperfusor | 0.24 | mg/h |
|  | opioid: Sufenta mite | 0.01 | mg |
|  | musc.relax.: Esmeron / Rocuronium | 10 | mg |
|  | cardio: Arterenolperfusor | 0.12 | mg/h |
|  | cardio: Arterenolperfusor | 0.06 | mg/h |
|  | musc.relax.: Esmeron / Rocuronium | 10 | mg |
|  | opioid: Sufenta mite | 0.01 | mg |
|  | inhal.anesth.: Sevofluran / Sevorane | 2.2 | % |
|  | cardio: Arterenolperfusor | 0.12 | mg/h |
|  | cardio: Arterenolperfusor | 0.18 | mg/h |
|  | cardio: Arterenolperfusor | 0.12 | mg/h |
|  | cardio: Arterenolperfusor | 0.24 | mg/h |
|  | opioid: Sufenta mite | 0.01 | mg |
|  | cardio: Arterenolperfusor | 0.06 | mg/h |
|  | cardio: Arterenolperfusor | 0.18 | mg/h |
|  | opioid: Sufenta mite | 0.03 | mg |
|  | cardio: Arterenolperfusor | 0.06 | mg/h |
|  | inhal.anesth.: Sevofluran / Sevorane | 2 | % |
|  | cardio: Arterenolperfusor | 0.18 | mg/h |
|  | musc.relax.: Esmeron / Rocuronium | 10 | mg |
|  | cardio: Arterenolperfusor | 0.06 | mg/h |
|  | cardio: Arterenolperfusor | 0.12 | mg/h |
|  | cardio: Arterenolperfusor | 0.18 | mg/h |
|  | cardio: Arterenolperfusor | 0.24 | mg/h |
|  | cardio: Arterenolperfusor | 0.12 | mg/h |
|  | opioid: Sufenta mite | 0.01 | mg |
| NL29 | sedat.&analg.: Dalmadorm | 30 | mg |
|  | sedat.&analg.: Dormicum | 7.5 | mg |
|  | infus.&blood: Sterofundin 1/1 E | 2500 | ml |
|  | opioid: Sufenta mite | 0.04 | mg |
|  | sedat.&analg.: Propofol 1 % | 160 | mg |
|  | musc.relax.: Esmeron / Rocuronium | 40 | mg |
|  | inhal.anesth.: Sevofluran / Sevorane | 1.4 | % |
|  | cardio: Arterenol | 0.05 | mg |
|  | opioid: Sufenta mite | 0.01 | mg |
|  | cardio: Arterenolperfusor | 0.24 | mg/h |
|  | inhal.anesth.: Sevofluran / Sevorane | 1.7 | % |
|  | antibiosis: Cefuroxime | 1500 | mg |
|  | antibiosis: Metronidazole / Clont | 500 | mg |
|  | opioid: Sufenta mite | 0.02 | mg |
|  | musc.relax.: Esmeron / Rocuronium | 20 | mg |
|  | opioid: Sufenta mite | 0.01 | mg |
|  | cardio: Arterenolperfusor | 0.12 | mg/h |
|  | inhal.anesth.: Sevofluran / Sevorane | 1.8 | % |
|  | opioid: Sufenta mite | 0.01 | mg |
|  | opioid: Sufenta mite | 0.01 | mg |
|  | infus.&blood: Voluven | 1000 | ml |
|  | musc.relax.: Esmeron / Rocuronium | 20 | mg |
|  | cardio: Arterenolperfusor | 0.24 | mg/h |
|  | opioid: Sufenta mite | 0.01 | mg |
|  | cardio: Arterenolperfusor | 0.3 | mg/h |
|  | opioid: Sufenta mite | 0.02 | mg |
|  | cardio: Arterenolperfusor | 0.24 | mg/h |
|  | cardio: Arterenolperfusor | 0.3 | mg/h |
|  | opioid: Sufenta mite | 0.02 | mg |
|  | infus.&blood: Packed red blood cells | 1 | n/a |
|  | musc.relax.: Esmeron / Rocuronium | 20 | mg |
|  | cardio: Arterenolperfusor | 0.42 | mg/h |
|  | cardio: Arterenolperfusor | 0.48 | mg/h |
|  | opioid: Sufenta mite | 0.02 | mg |
| NL30 | infus.&blood: Packed red blood cells | 1 | n/a |
|  | sedat.&analg.: Dormicum | 7.5 | mg |
|  | infus.&blood: Sterofundin 1/1 E | 3000 | ml |
|  | opioid: Sufenta mite | 0.04 | mg |
|  | sedat.&analg.: Propofol 1 % | 120 | mg |
|  | musc.relax.: Esmeron / Rocuronium | 40 | mg |
|  | sedat.&analg.: Propofol 1 % | 30 | mg |
|  | antibiosis: Cefuroxime | 1500 | mg |
|  | cardio: Arterenol | 0.01 | mg |
|  | inhal.anesth.: Sevofluran / Sevorane | 1.4 | mg |
|  | cardio: Arterenol | 0.01 | mg |
|  | antibiosis: Metronidazole / Clont | 500 | mg |
|  | cardio: Arterenol | 0.01 | mg |
|  | opioid: Sufenta mite | 0.01 | mg |
|  | cardio: Arterenolperfusor | 0.3 | mg/h |
|  | cardio: Arterenolperfusor | 0.18 | mg/h |
|  | opioid: Sufenta mite | 0.02 | mg |
|  | musc.relax.: Esmeron / Rocuronium | 20 | mg |
|  | inhal.anesth.: Sevofluran / Sevorane | 1.6 | mg |
|  | opioid: Sufenta mite | 0.02 | mg |
|  | cardio: Arterenolperfusor | 0.12 | mg/h |
|  | inhal.anesth.: Sevofluran / Sevorane | 1.9 | mg |
|  | opioid: Sufenta mite | 0.02 | mg |
|  | musc.relax.: Esmeron / Rocuronium | 10 | mg |
|  | cardio: Arterenolperfusor | 0.3 | mg/h |
|  | opioid: Sufenta mite | 0.01 | mg |
|  | cardio: Arterenolperfusor | 0.12 | mg/h |
|  | inhal.anesth.: Sevofluran / Sevorane | 2 | mg |
|  | opioid: Sufenta mite | 0.02 | mg |
|  | cardio: Arterenolperfusor | 0.12 | mg/h |
|  | cardio: Arterenolperfusor | 0.24 | mg/h |
|  | infus.&blood: Voluven | 500 | ml |
|  | cardio: Arterenolperfusor | 0.36 | mg/h |
|  | opioid: Sufenta mite | 0.01 | mg |
|  | cardio: Arterenolperfusor | 0.42 | mg/h |
|  | sedat.&analg.: Novamin / Novalgin | 1000 | mg |
|  | cardio: Arterenolperfusor | 0.48 | mg/h |
|  | infus.&blood: Hydroxyethyl starch | 500 | ml |
| NL31 | sedat.&analg.: Dormicum | 7.5 | mg |
|  | sedat.&analg.: Dormicum | 7.5 | mg |
|  | opioid: Sufenta mite | 0.04 | mg |
|  | sedat.&analg.: Propofol 1 % | 200 | mg |
|  | infus.&blood: Ringer solution | 1500 | ml |
|  | musc.relax.: Esmeron / Rocuronium | 50 | mg |
|  | inhal.anesth.: Sevofluran / Sevorane | 1.4 | % |
|  | sedat.&analg.: Propofol 1 % | 100 | mg |
|  | cardio: Arterenolperfusor | 0.18 | mg/h |
|  | cardio: Arterenol | 0.04 | mg |
|  | infus.&blood: Voluven | 500 | ml |
|  | cardio: Arterenolperfusor | 0.3 | mg/h |
|  | antibiosis: Cefuroxime | 1500 | mg |
|  | opioid: Sufenta mite | 0.01 | mg |
|  | inhal.anesth.: Sevofluran / Sevorane | 1.9 | % |
|  | musc.relax.: Esmeron / Rocuronium | 20 | mg |
|  | opioid: Sufenta mite | 0.02 | mg |
|  | opioid: Sufenta mite | 0.02 | mg |
|  | cardio: Arterenolperfusor | 0.12 | mg/h |
|  | cardio: Arterenolperfusor | 0.18 | mg/h |
|  | cardio: Arterenolperfusor | 0.24 | mg/h |
|  | opioid: Sufenta mite | 0.015 | mg |
|  | cardio: Arterenolperfusor | 0.3 | mg/h |
|  | opioid: Sufenta mite | 0.01 | mg |
|  | cardio: Arterenolperfusor | 0.42 | mg/h |
|  | cardio: Arterenolperfusor | 0.48 | mg/h |
| NL32 | sedat.&analg.: Dormicum | 7.5 | mg |
|  | opioid: Sufenta mite | 0.05 | mg |
|  | infus.&blood: Sterofundin 1/1 E | 2000 | ml |
|  | sedat.&analg.: Propofol 1 % | 200 | mg |
|  | musc.relax.: Esmeron / Rocuronium | 50 | mg |
|  | inhal.anesth.: Sevofluran / Sevorane | 2.1 | mg |
|  | antibiosis: Cefuroxime | 1500 | mg |
|  | antibiosis: Metronidazole / Clont | 500 | mg |
|  | cardio: Arterenol | 0.01 | mg |
|  | cardio: Arterenolperfusor | 0.18 | mg/h |
|  | cardio: Arterenol | 0.01 | mg |
|  | cardio: Arterenolperfusor | 0.3 | mg/h |
|  | inhal.anesth.: Sevofluran / Sevorane | 2 | mg |
|  | opioid: Sufenta mite | 0.03 | mg |
|  | musc.relax.: Esmeron / Rocuronium | 40 | mg |
|  | cardio: Arterenolperfusor | 0.24 | mg/h |
|  | cardio: Arterenolperfusor | 0.12 | mg/h |
|  | opioid: Sufenta mite | 0.02 | mg |
|  | cardio: Arterenolperfusor | 0.06 | mg/h |
|  | cardio: Arterenolperfusor | 0.12 | mg/h |
|  | musc.relax.: Esmeron / Rocuronium | 10 | mg |
|  | cardio: Arterenolperfusor | 0.06 | mg/h |
|  | cardio: Arterenolperfusor | 0.12 | mg/h |
|  | musc.relax.: Esmeron / Rocuronium | 10 | mg |
|  | opioid: Sufenta mite | 0.01 | mg |
|  | cardio: Arterenolperfusor | 0.04 | mg/h |
|  | infus.&blood: Hydroxyethyl starch | 500 | ml |
|  | cardio: Arterenolperfusor | 0.12 | mg/h |
|  | opioid: Sufenta mite | 0.02 | mg |
|  | musc.relax.: Esmeron / Rocuronium | 10 | mg |
|  | musc.relax.: Esmeron / Rocuronium | 10 | mg |
|  | opioid: Sufenta mite | 0.02 | mg |
|  | cardio: Arterenolperfusor | 0.3 | mg/h |
| NL33 | sedat.&analg.: Dormicum | 3.75 | mg |
|  | sedat.&analg.: Propofol 1 % | 100 | mg |
|  | infus.&blood: Sterofundin 1/1 E | 3000 | ml |
|  | musc.relax.: Esmeron / Rocuronium | 40 | mg |
|  | opioid: Sufenta mite | 0.04 | mg |
|  | cardio: Arterenol | 0.01 | mg |
|  | sedat.&analg.: Propofol 1 % | 50 | mg |
|  | inhal.anesth.: Sevofluran / Sevorane | 0.5 | % |
|  | cardio: Arterenol | 6.01 | mg |
|  | cardio: Arterenol | 0.02 | mg |
|  | cardio: Arterenol | 0.01 | mg |
|  | cardio: Arterenolperfusor | 0.36 | mg/h |
|  | opioid: Sufenta mite | 0.02 | mg |
|  | antibiosis: Cefuroxime | 1500 | mg |
|  | cardio: Atropine | 0.5 | mg |
|  | cardio: Arterenolperfusor | 0.12 | mg/h |
|  | inhal.anesth.: Sevofluran / Sevorane | 1.1 | % |
|  | musc.relax.: Esmeron / Rocuronium | 10 | mg |
|  | antibiosis: Metronidazole / Clont | 500 | mg |
|  | inhal.anesth.: Sevofluran / Sevorane | 1.9 | % |
|  | opioid: Sufenta mite | 0.02 | mg |
|  | infus.&blood: Potassium chloride 7.45% | 20 | mval |
|  | musc.relax.: Esmeron / Rocuronium | 20 | mg |
|  | opioid: Sufenta mite | 0.02 | mg |
|  | cardio: Arterenolperfusor | 0.18 | mg/h |
|  | cardio: Arterenolperfusor | 0.3 | mg/h |
|  | inhal.anesth.: Sevofluran / Sevorane | 1.8 | % |
|  | cardio: Arterenolperfusor | 0.24 | mg/h |
|  | cardio: Arterenolperfusor | 0.3 | mg/h |
|  | inhal.anesth.: Sevofluran / Sevorane | 1.9 | % |
|  | opioid: Sufenta mite | 0.02 | mg |
|  | cardio: Arterenolperfusor | 0.6 | mg/h |
|  | infus.&blood: Voluven | 500 | ml |
| NL34 | sedat.&analg.: Dormicum | 7.5 | mg |
|  | sedat.&analg.: Propofol 1 % | 200 | mg |
|  | opioid: Sufenta mite | 0.05 | mg |
|  | infus.&blood: Sterofundin 1/1 E | 3000 | ml |
|  | musc.relax.: Esmeron / Rocuronium | 50 | mg |
|  | sedat.&analg.: Propofol 1 % | 50 | mg |
|  | antibiosis: Cefuroxime | 1500 | mg |
|  | cardio: Arterenol | 0.01 | mg |
|  | inhal.anesth.: Sevofluran / Sevorane | 1.8 | % |
|  | cardio: Arterenol | 0.01 | mg |
|  | cardio: Arterenolperfusor | 0.3 | mg/h |
|  | cardio: Arterenol | 0.01 | mg |
|  | antibiosis: Metronidazole / Clont | 500 | mg |
|  | cardio: Arterenolperfusor | 0.48 | mg/h |
|  | opioid: Sufenta mite | 0.05 | mg |
|  | musc.relax.: Esmeron / Rocuronium | 50 | mg |
|  | cardio: Arterenolperfusor | 0.36 | mg/h |
|  | inhal.anesth.: Sevofluran / Sevorane | 2 | % |
|  | opioid: Sufenta mite | 0.03 | mg |
|  | cardio: Arterenolperfusor | 0.18 | mg/h |
|  | cardio: Arterenolperfusor | 0.3 | mg/h |
|  | musc.relax.: Esmeron / Rocuronium | 10 | mg |
|  | opioid: Sufenta mite | 0.02 | mg |
|  | cardio: Arterenolperfusor | 0.6 | mg/h |
|  | cardio: Arterenolperfusor | 0.48 | mg/h |
|  | cardio: Arterenolperfusor | 0.36 | mg/h |
|  | musc.relax.: Esmeron / Rocuronium | 10 | mg |
|  | opioid: Sufenta mite | 0.02 | mg |
|  | opioid: Sufenta mite | 0.01 | mg |
|  | cardio: Arterenolperfusor | 0.48 | mg/h |
|  | cardio: Arterenolperfusor | 0.36 | mg/h |
|  | opioid: Sufenta mite | 0.01 | mg |
|  | cardio: Arterenolperfusor | 0.24 | mg/h |
|  | cardio: Arterenolperfusor | 0.48 | mg/h |
|  | opioid: Sufenta mite | 0.01 | mg |
|  | cardio: Arterenolperfusor | 0.36 | mg/h |
|  | musc.relax.: Esmeron / Rocuronium | 10 | mg |
|  | opioid: Sufenta mite | 0.02 | mg |
|  | cardio: Arterenolperfusor | 0.6 | mg/h |
|  | cardio: Arterenolperfusor | 1.08 | mg/h |
|  | cardio: Arterenolperfusor | 0.9 | mg/h |
|  | cardio: Arterenolperfusor | 0.78 | mg/h |
|  | opioid: Sufenta mite | 0.01 | mg |
|  | cardio: Arterenolperfusor | 0.9 | mg/h |
|  | cardio: Arterenolperfusor | 0.78 | mg/h |
|  | opioid: Sufenta mite | 0.02 | mg |
|  | antibiosis: Cefuroxime | 1500 | mg |
|  | cardio: Arterenolperfusor | 0.9 | mg/h |
|  | infus.&blood: Voluven | 500 | ml |
|  | antibiosis: Metronidazole / Clont | 500 | mg |
| NL36 | sedat.&analg.: Dalmadorm | 15 | mg |
|  | gastrointest.&antihist.: Zantic | 300 | mg |
|  | gastrointest.&antihist.: Zantic | 150 | mg |
|  | sedat.&analg.: Dormicum | 3.75 | mg |
|  | sedat.&analg.: Propofol 1 % | 200 | mg |
|  | infus.&blood: Sterofundin 1/1 E | 1000 | ml |
|  | opioid: Sufenta mite | 0.03 | mg |
|  | musc.relax.: Esmeron / Rocuronium | 30 | mg |
|  | antibiosis: Cefuroxime | 1500 | mg |
|  | inhal.anesth.: Sevofluran / Sevorane | 1 | % |
|  | cardio: Arterenolperfusor | 0.18 | mg/h |
|  | inhal.anesth.: Sevofluran / Sevorane | 1.6 | % |
|  | antibiosis: Metronidazole / Clont | 500 | mg |
|  | opioid: Sufenta mite | 0.02 | mg |
|  | musc.relax.: Esmeron / Rocuronium | 30 | mg |
|  | cardio: Arterenolperfusor | 0.24 | mg/h |
|  | inhal.anesth.: Sevofluran / Sevorane | 1.7 | % |
|  | cardio: Arterenolperfusor | 0.18 | mg/h |
|  | inhal.anesth.: Sevofluran / Sevorane | 1.9 | % |
|  | musc.relax.: Esmeron / Rocuronium | 10 | mg |
|  | opioid: Sufenta mite | 0.015 | mg |
|  | cardio: Arterenolperfusor | 0.3 | mg/h |
| NL37 | sedat.&analg.: Dormicum | 7.5 | mg |
|  | antibiosis: Metronidazole / Clont | 500 | mg |
|  | sedat.&analg.: Propofol 1 % | 200 | mg |
|  | opioid: Sufenta / Sufentanil | 0.01 | mg |
|  | antibiosis: Basocef / Cefazolin / Elzogram | 2000 | mg |
|  | sedat.&analg.: Propofolperfusor | 20 | mg/h |
|  | musc.relax.: Norcuron / Vecuronium | 10 | mg |
|  | opioid: Sufenta / Sufentanil | 0.02 | mg |
|  | infus.&blood: Ringeracetat | 500 | ml |
|  | musc.relax.: Norcuron / Vecuronium | 2.5 | mg |
|  | infus.&blood: Hydroxyethyl starch | 500 | ml |
|  | musc.relax.: Norcuron / Vecuronium | 5 | mg |
|  | cardio: Arterenol | 0.08 | mg |
|  | diuretic&corticost.: Furosemide / Lasix | 10 | mg |
|  | opioid: Sufenta / Sufentanil | 0.02 | mg |
|  | musc.relax.: Norcuron / Vecuronium | 2.5 | mg |
|  | cardio: Arterenol | 0.32 | mg |
|  | cardio: Atropine | 0.75 | mg |
|  | cardio: Alupent | 0.2 | mg |
|  | diuretic&corticost.: Furosemide / Lasix | 10 | mg |
|  | cardio: Arterenol | 0.2 | mg |
|  | opioid: Sufenta / Sufentanil | 0.02 | mg |
|  | cardio: Arterenol | 0.08 | mg |
|  | opioid: Sufenta / Sufentanil | 0.015 | mg |
|  | diuretic&corticost.: Furosemide / Lasix | 10 | mg |
|  | cardio: Arterenol | 0.2 | mg |
|  | cardio: Arterenol | 0.32 | mg |
|  | cardio: Arterenol | 0.6 | mg |
|  | cardio: Arterenol | 0.28 | mg |
|  | opioid: Sufenta / Sufentanil | 0.015 | mg |
|  | musc.relax.: Norcuron / Vecuronium | 2.5 | mg |
| NL38 | sedat.&analg.: Tranxilium | 25 | mg |
|  | sedat.&analg.: Dormicum | 7.5 | mg |
|  | antibiosis: Basocef / Cefazolin / Elzogram | 2000 | mg |
|  | loc.anesth.: Scandicain | 20 | mg |
|  | infus.&blood: Hydroxyethyl starch | 500 | ml |
|  | cardio: Suprarenin | 10 | mg |
|  | opioid: Sufenta / Sufentanil | 0.025 | mg |
|  | sedat.&analg.: Propofol 1 % | 250 | mg |
|  | musc.relax.: Norcuron / Vecuronium | 10 | mg |
|  | inhal.anesth.: Sevofluran / Sevorane | 0.9 | % |
|  | cardio: Suprarenin | 10 | mg |
|  | infus.&blood: Ringeracetat | 500 | ml |
|  | antibiosis: Metronidazole / Clont | 500 | mg |
|  | cardio: Suprarenin | 10 | mg |
|  | infus.&blood: Potassium chloride 7.45% | 20 | mval |
|  | loc.anesth.: Naropin | 75 | mg |
|  | cardio: Arterenol | 5 | mg |
|  | cardio: Arterenol | 12 | mg |
|  | cardio: Arterenol | 15 | mg |
| NL40 | sedat.&analg.: Dalmadorm | 30 | mg |
|  | sedat.&analg.: Arcoxia | 120 | mg |
|  | sedat.&analg.: Dormicum | 7.5 | mg |
|  | sedat.&analg.: Propofol 1 % | 250 | mg |
|  | infus.&blood: Ringeracetat | 500 | ml |
|  | opioid: Sufenta mite | 0.04 | mg |
|  | musc.relax.: Esmeron / Rocuronium | 50 | mg |
|  | antibiosis: Metronidazole / Clont | 500 | mg |
|  | inhal.anesth.: Sevofluran / Sevorane | 1.4 | % |
|  | antibiosis: Cefuroxime | 1500 | mg |
|  | opioid: Sufenta mite | 0.01 | mg |
|  | cardio: Arterenol | 0.03 | mg |
|  | inhal.anesth.: Sevofluran / Sevorane | 2 | % |
|  | cardio: Arterenolperfusor | 0.36 | mg/h |
|  | cardio: Atropine | 0.5 | mg |
|  | opioid: Sufenta mite | 0.02 | mg |
|  | infus.&blood: Hydroxyethyl starch | 1000 | ml |
|  | opioid: Sufenta mite | 0.01 | mg |
|  | opioid: Sufenta mite | 0.02 | mg |
|  | musc.relax.: Esmeron / Rocuronium | 20 | mg |
|  | cardio: Arterenolperfusor | 0.24 | mg/h |
|  | opioid: Sufenta mite | 0.015 | mg |
|  | infus.&blood: Sterofundin 1/1 E | 1000 | ml |
|  | cardio: Arterenolperfusor | 0.42 | mg/h |
| NL41 | infus.&blood: Packed red blood cells | 2 | n/a |
|  | infus.&blood: Fresh Frozen Plasma (FFP) | 1 | n/a |
|  | sedat.&analg.: Tranxilium | 50 | mg |
|  | sedat.&analg.: Dormicum | 7.5 | mg |
|  | antibiosis: Metronidazole / Clont | 500 | mg |
|  | antibiosis: Basocef / Cefazolin / Elzogram | 2000 | mg |
|  | loc.anesth.: Scandicain | 30 | mg |
|  | musc.relax.: Norcuron / Vecuronium | 10 | mg |
|  | musc.relax.: Pantolax / Lysthenon / Succinylcholine | 100 | mg |
|  | sedat.&analg.: Propofol 1 % | 300 | mg |
|  | opioid: Sufenta / Sufentanil | 0.05 | mg |
|  | inhal.anesth.: Sevofluran / Sevorane | 2.4 | % |
|  | cardio: Akrinor | 50 | mg |
|  | loc.anesth.: Naropin | 37.5 | mg |
|  | opioid: Sufenta / Sufentanil | 0.01 | mg |
|  | cardio: Arterenolperfusor | 0.12 | mg/h |
|  | sedat.&analg.: Propofol 1 % | 50 | mg |
|  | opioid: Sufenta / Sufentanil | 0.01 | mg |
|  | cardio: Arterenolperfusor | 0.24 | mg/h |
|  | opioid: Sufenta / Sufentanil | 0.02 | mg |
|  | musc.relax.: Norcuron / Vecuronium | 9 | mg |
|  | cardio: Arterenolperfusor | 0.24 | mg/h |
|  | opioid: Sufenta / Sufentanil | 0.01 | mg |
|  | cardio: Arterenolperfusor | 0.32 | mg/h |
|  | loc.anesth.: Naropin | 30 | mg |
|  | cardio: Arterenolperfusor | 0.56 | mg/h |
|  | opioid: Sufenta / Sufentanil | 0.02 | mg |
|  | cardio: Arterenolperfusor | 0.8 | mg/h |
|  | infus.&blood: Packed red blood cells | 1 | n/a |
|  | cardio: Arterenolperfusor | 0.32 | mg/h |
|  | inhal.anesth.: Sevofluran / Sevorane | 2.8 | % |
|  | diuretic&corticost.: Furosemide / Lasix | 5 | mg |
| NL42 | sedat.&analg.: Dormicum | 3.75 | mg |
|  | cardio: Arterenolperfusor | 0.18 | mg/h |
|  | antibiosis: Metronidazole / Clont | 500 | mg |
|  | antibiosis: Cefuroxime | 1500 | mg |
|  | infus.&blood: Sterofundin 1/1 E | 1500 | ml |
|  | cardio: Arterenolperfusor | 0.36 | mg/h |
|  | musc.relax.: Esmeron / Rocuronium | 40 | mg |
|  | sedat.&analg.: Propofol 1 % | 160 | mg |
|  | opioid: Sufenta mite | 0.05 | mg |
|  | inhal.anesth.: Sevofluran / Sevorane | 1.6 | % |
|  | inhal.anesth.: Sevofluran / Sevorane | 2 | % |
|  | musc.relax.: Esmeron / Rocuronium | 10 | mg |
|  | opioid: Sufenta mite | 0.01 | mg |
|  | cardio: Arterenolperfusor | 0.3 | mg/h |
|  | musc.relax.: Esmeron / Rocuronium | 10 | mg |
|  | opioid: Sufenta mite | 0.03 | mg |
|  | cardio: Arterenolperfusor | 0.18 | mg/h |
|  | cardio: Arterenolperfusor | 0.12 | mg/h |
|  | opioid: Sufenta mite | 0.01 | mg |
|  | inhal.anesth.: Sevofluran / Sevorane | 2.1 | % |
|  | opioid: Sufenta mite | 0.01 | mg |
|  | cardio: Arterenolperfusor | 0.06 | mg/h |
| NL43 | sedat.&analg.: Dormicum | 3.75 | mg |
|  | opioid: Sufenta mite | 0.035 | mg |
|  | sedat.&analg.: Propofol 1 % | 150 | mg |
|  | cardio: Arterenol | 0.01 | mg |
|  | antibiosis: Cefuroxime | 1500 | mg |
|  | musc.relax.: Esmeron / Rocuronium | 40 | mg |
|  | antibiosis: Metronidazole / Clont | 500 | mg |
|  | cardio: Arterenol | 0.015 | mg |
|  | infus.&blood: Sterofundin 1/1 E | 1500 | ml |
|  | inhal.anesth.: Sevofluran / Sevorane | 1.5 | % |
|  | cardio: Arterenol | 0.02 | mg |
|  | cardio: Arterenol | 0.015 | mg |
|  | infus.&blood: Voluven | 500 | ml |
|  | cardio: Arterenolperfusor | 0.6 | mg/h |
|  | inhal.anesth.: Sevofluran / Sevorane | 1 | % |
|  | cardio: Arterenolperfusor | 0.36 | mg/h |
|  | cardio: Arterenolperfusor | 0.24 | mg/h |
|  | cardio: Arterenolperfusor | 0.12 | mg/h |
|  | opioid: Sufenta mite | 0.03 | mg |
|  | musc.relax.: Esmeron / Rocuronium | 10 | mg |
|  | musc.relax.: Esmeron / Rocuronium | 30 | mg |
|  | opioid: Sufenta mite | 0.02 | mg |
|  | inhal.anesth.: Sevofluran / Sevorane | 1.3 | % |
|  | cardio: Clonidine / Catapresan | 0.075 | mg |
|  | cardio: Arterenolperfusor | 0.06 | mg/h |
|  | opioid: Sufenta mite | 0.015 | mg |
|  | musc.relax.: Esmeron / Rocuronium | 20 | mg |
|  | cardio: Arterenolperfusor | 0.18 | mg/h |
|  | cardio: Arterenolperfusor | 0.12 | mg/h |

**Supplementary Table S2.** List of identified metabolites.The MSI levels of identification and the compound IDs derived from the Human Metabolome Database (HMDB) are provided.

| Metabolite | MSI level of identification | Database | Compound ID |
| --- | --- | --- | --- |
| Acetate (Acetic acid) | 1 | HMDB | HMDB00042 |
| Acetoacetate (Acetoacetic acid) | 1 | HMDB | HMDB00060 |
| N-Acetylcysteine | 1 | HMDB | HMDB01890 |
| Alanine | 1 | HMDB | HMDB00161 |
| Arginine | 1 | HMDB | HMDB00517 |
| Cefuroxime | 2 | HMDB | HMDB15052 |
| Citrate (Citric acid) | 1 | HMDB | HMDB00094 |
| Creatine | 1 | HMDB | HMDB00064 |
| Creatinine | 1 | HMDB | HMDB00562 |
| Dimethylamine | 1 | HMDB | HMDB00087 |
| Formate (Formic acid) | 1 | HMDB | HMDB00142 |
| Glutamate (Glutamic acid) | 1 | HMDB | HMDB03339 |
| Glutamine | 1 | HMDB | HMDB00641 |
| Histidine | 1 | HMDB | HMDB00177 |
| 3-Hydroxyisobutirate  (3-Hydroxyisobutirc acid) | 1 | HMDB | HMDB00023 |
| Isoleucine | 1 | HMDB | HMDB00172 |
| Lactate (Lactic acid) | 1 | HMDB | HMDB00190 |
| Leucine | 1 | HMDB | HMDB00687 |
| Phenylalanine | 1 | HMDB | HMDB00159 |
| Tyrosine | 1 | HMDB | HMDB00158 |
| Valine | 1 | HMDB | HMDB00883 |

**Supplementary Figures**

**
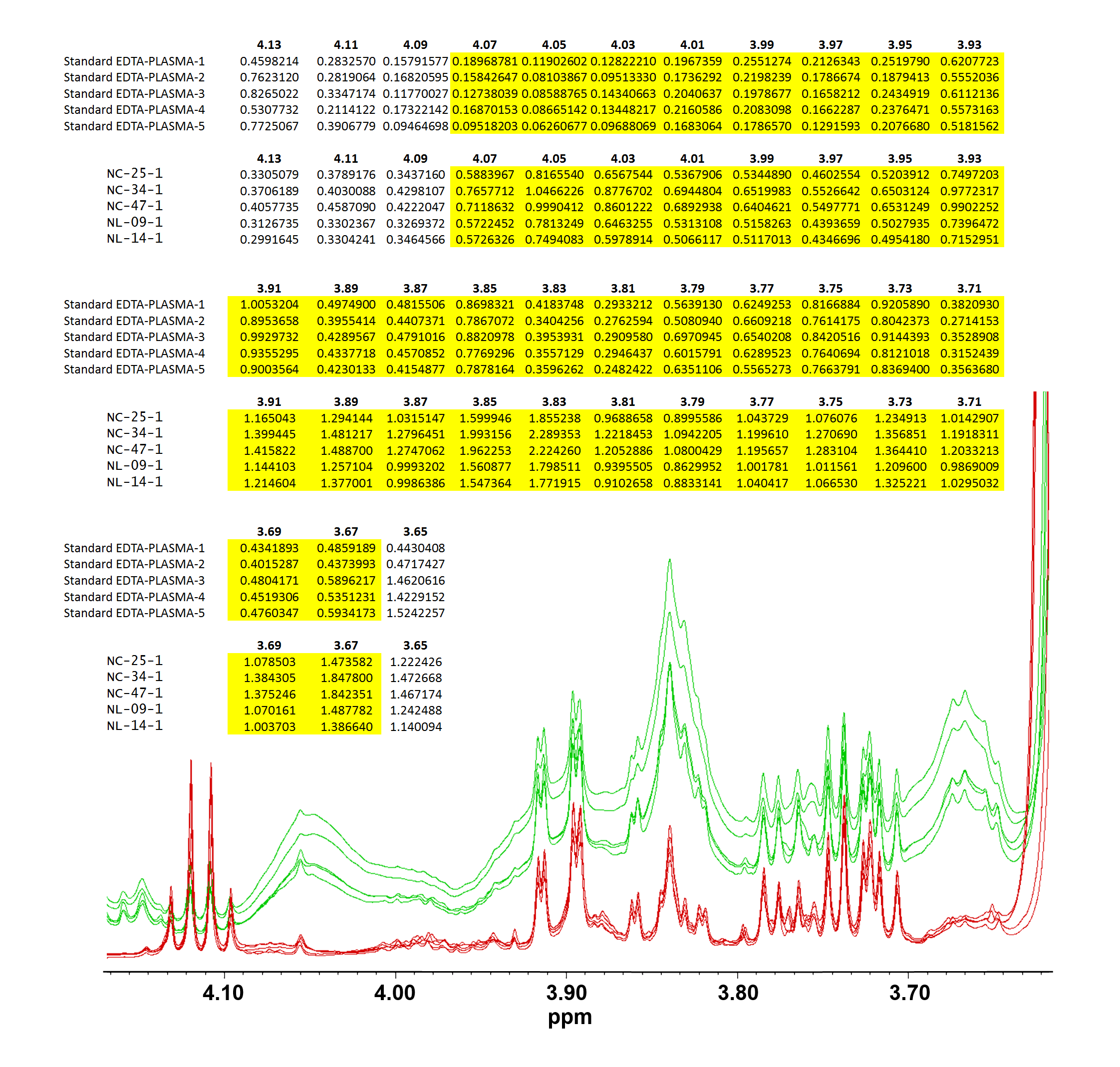
**

**Supplementary Figure S1.** Comparison of the CPMG spectra of 5 “normal” EDTA-plasma samples (red traces) and 5 Ficoll-EDTA-plasma samples of CRC patients (green traces), in the 3.60-4.20 ppm spectral area. The broad and intense absorbtions due to Ficoll contamination from 3.30 to 4.64 ppm are cleary visible in the green traces; contaminant resonances have different relative intensities in different samples. The “bucket table” for each spectrum in the most divergent spectral region is provided in the top panel; buckets with the largest differences between the two sets of spectra are highlighted in yellow.

**
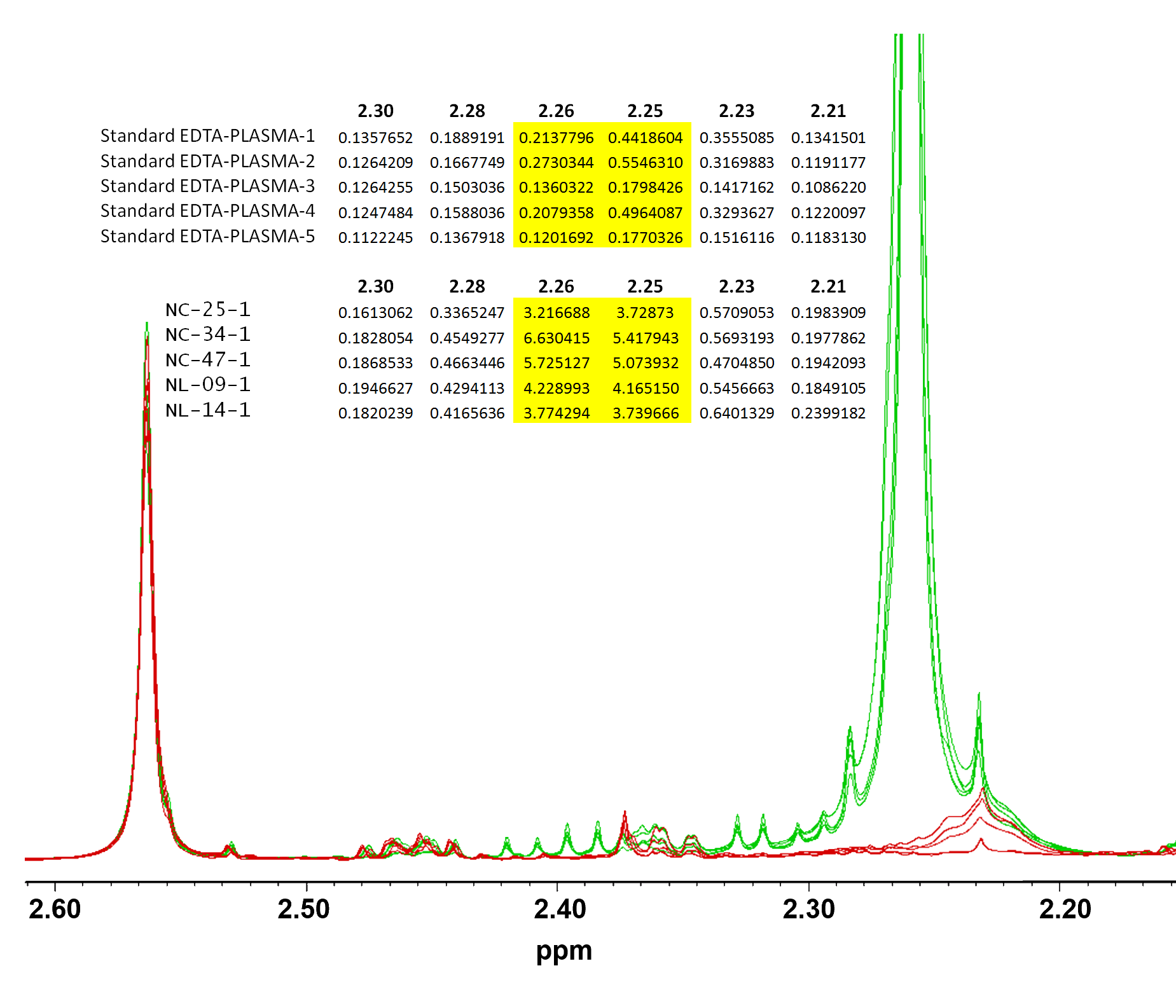
**

**Supplementary Figure S2.** Comparison of the CPMG spectra of 5 “normal” EDTA-plasma samples (red traces) and 5 Ficoll-EDTA-plasma samples of CRC patients (green traces), in the 2.20-2.60 ppm spectral area. The broad and intense absorbtions due to Ficoll contamination around 2.26 ppm are cleary visible in the green traces; contaminant resonances have different relative intensities in different samples. The “bucket table” for each spectrum in the most divergent spectral region is provided in the top panel; buckets with the largest differences between the two sets of spectra are highlighted in yellow.

**
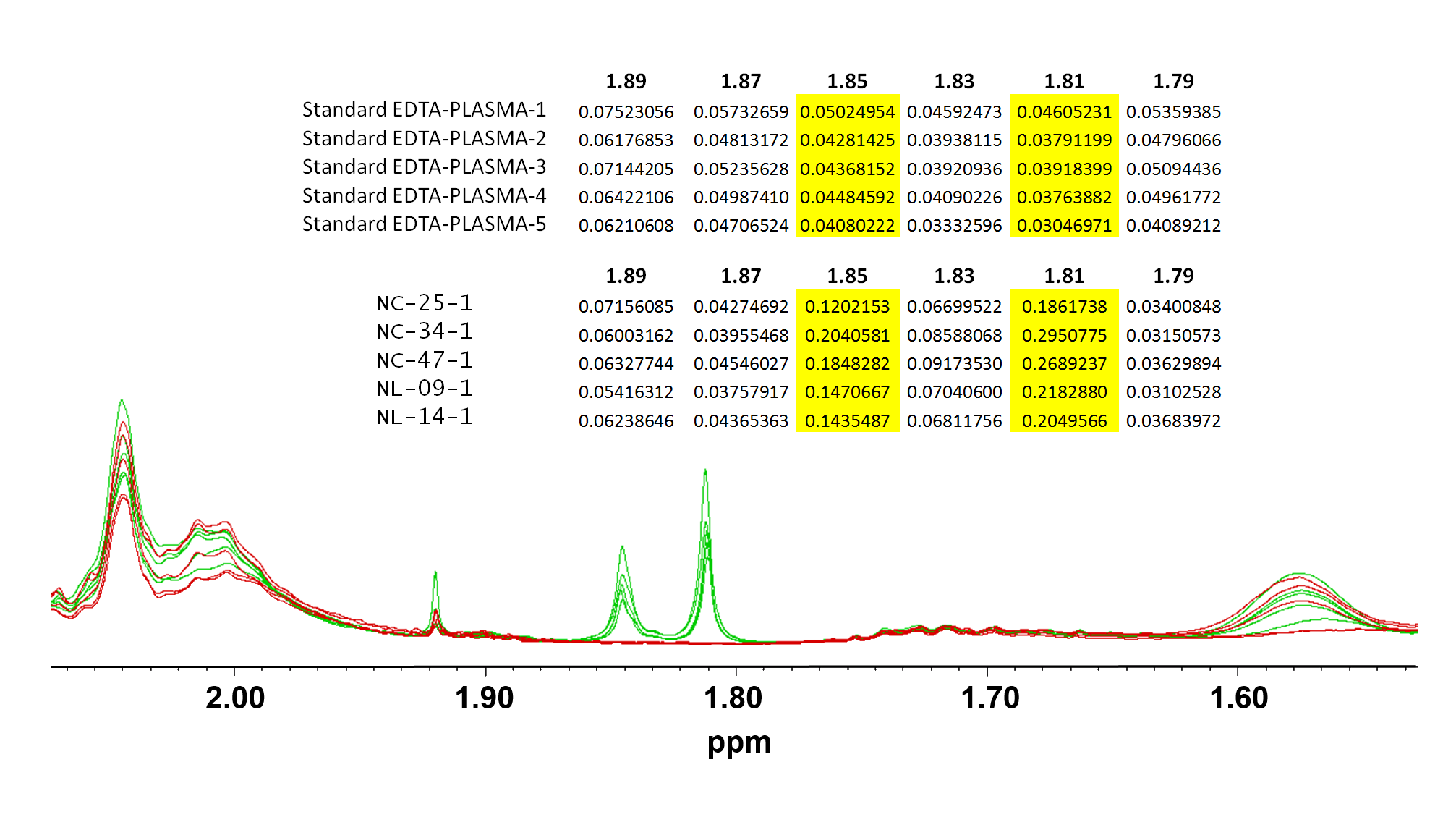
**

**Supplementary Figure S3.** Comparison of the CPMG spectra of 5 “normal” EDTA-plasma samples (red traces) and 5 Ficoll-EDTA-plasma samples of CRC patients (green traces), in the 1.60-2.00 ppm spectral area. The broad and intense absorbtions due to Ficoll contamination at 1.81 and 1.84 ppm are cleary visible in the green traces; contaminant resonances have different relative intensities in different samples. The “bucket table” for each spectrum in the most divergent spectral region is provided in the top panel; buckets with the largest differences between the two sets of spectra are highlighted in yellow.

**
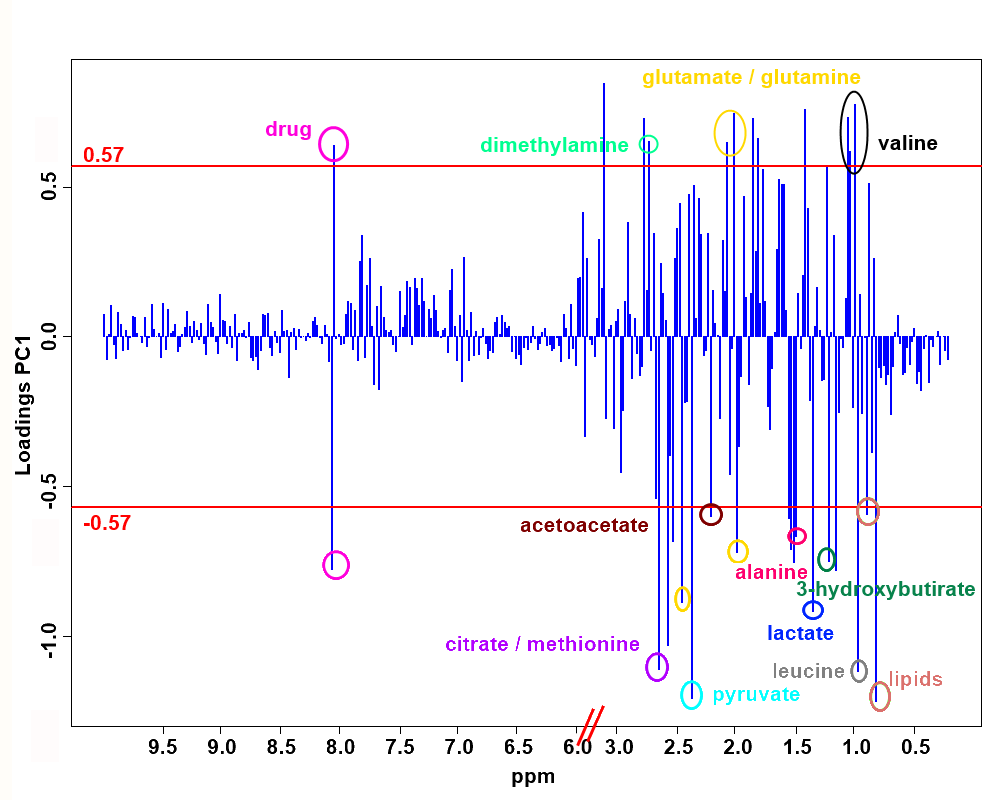
**

**Supplementary Figure S4.** PC1 loadings plot of PLS-CA discrimination of CRC and LC patients using pre-anesthesia samples. The significance threshold (red lines) was calculated considering "buckets" with a value beyond one standard deviation of their averages.


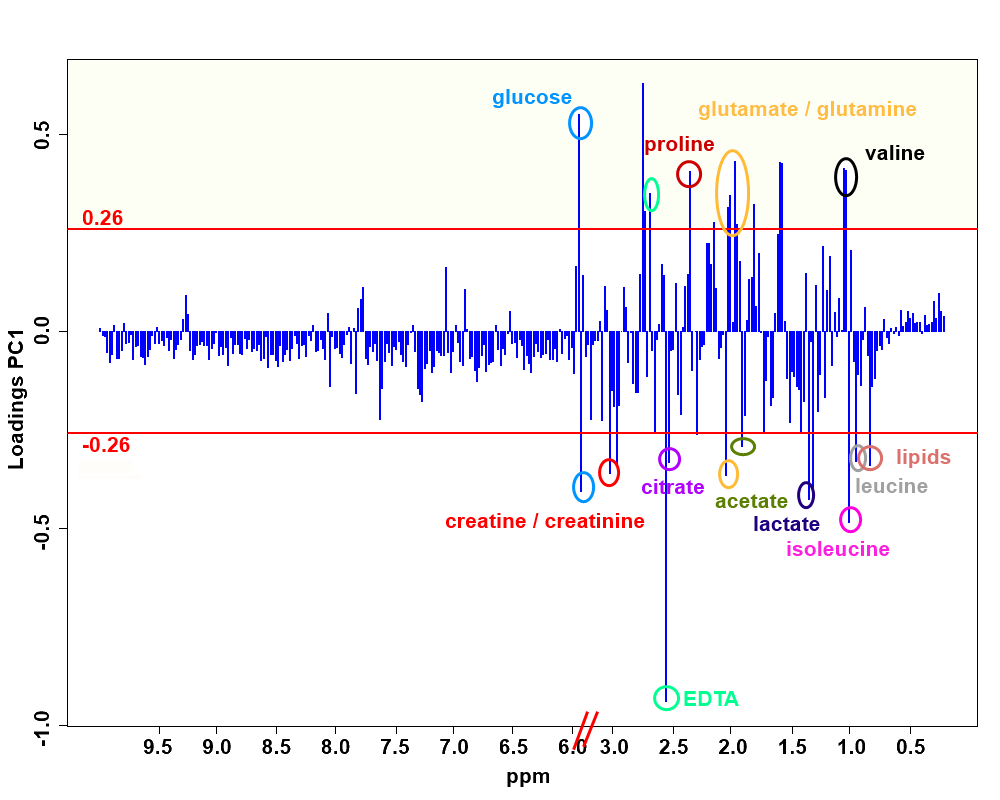


**Supplementary Figure S5.** PC1 loadings plot of PLS-CA discrimination of CRC and LC patients using post-anesthesia samples. The significance threshold (red lines) was calculated considering "buckets" with a value beyond one standard deviation of their averages.


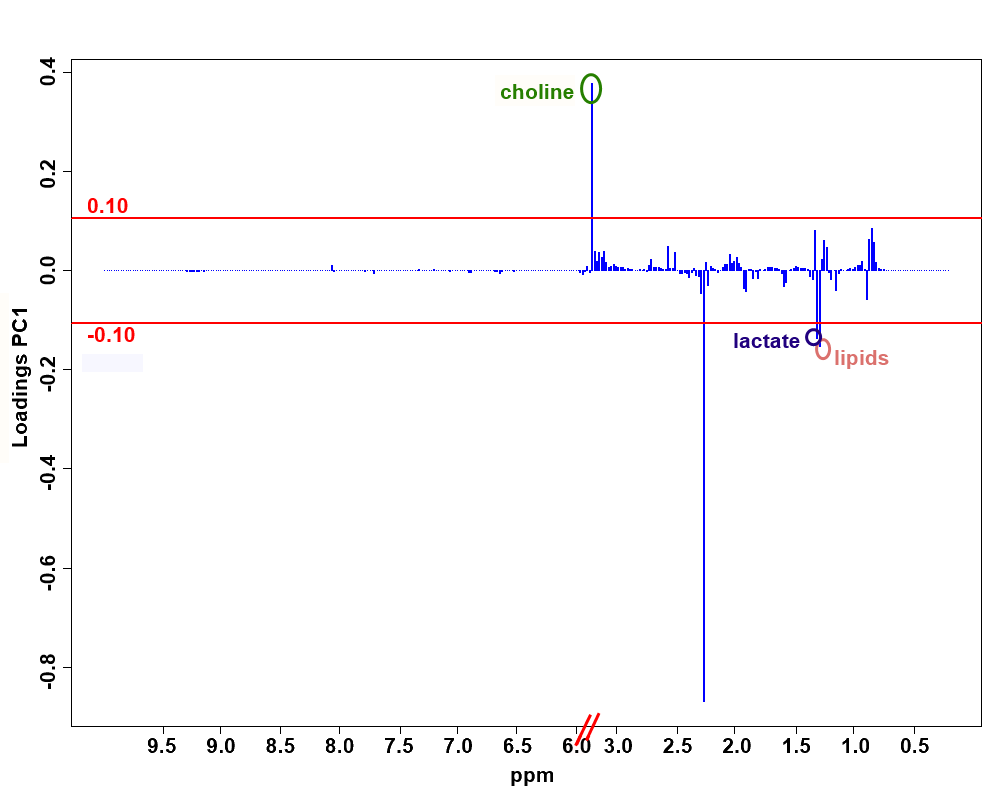


**Supplementary Figure S6.** PC1 loadings plot of Multilevel PLS discrimination of pre- and post-anesthesia using all CRC and LC samples. The significance threshold (red lines) was calculated considering "buckets" with a value beyond one standard deviation of their averages.


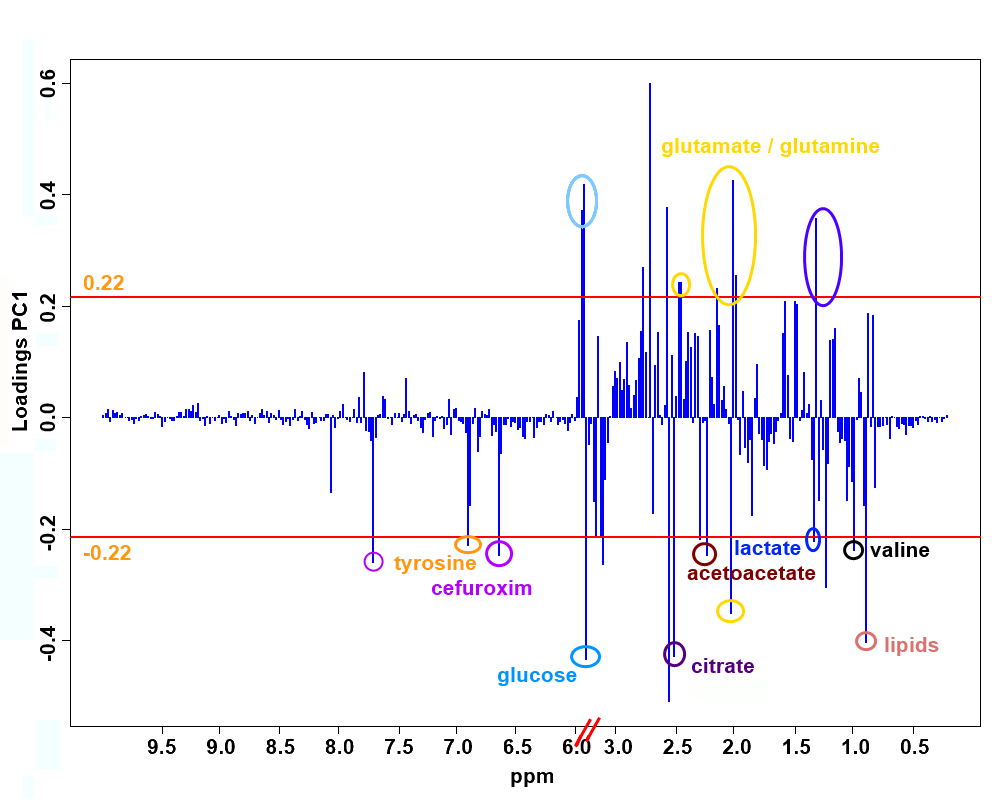


**Supplementary Figure S7.** PC1 loadings plot of PLS-CA discrimination of different anesthetics in post-anesthesia CRC samples (CRC patients treated with Etomidate vs CRC patients treated with Propofol 2%). The significance threshold (red lines) was calculated considering "buckets" with a value beyond one standard deviation of their averages.
